# Resolving Ternary Morphology for High-Performance Thickness-Insensitive Organic Solar Cells

**DOI:** 10.1021/acsami.5c17321

**Published:** 2025-11-28

**Authors:** Heng Liu, Yuhao Li, Zhaoyang Nie, Yuang Fu, Zhaozhao Bi, Shengtian Zhu, Lu Chen, Guilong Cai, Pok Fung Chan, Luo Huang, Luhang Xu, Chun-Jen Su, U-Ser Jeng, Guangye Zhang, Fei Huang, Wei Ma, Xian-Kai Chen, Yubin Ke, Man Chung Tang, Xinhui Lu

**Affiliations:** † Department of Physics, 26451 The Chinese University of Hong Kong, New Territories, Hong Kong 999077, China; ‡ Spallation Neutron Source Science Center, Institute of High Energy Physics, 71035 Chinese Academy of Sciences, Dongguan 523803, China; § Institute of Materials Research, Tsinghua Shenzhen International Graduate School, 12442 Tsinghua University, Shenzhen 518055, China; ∥ State Key Laboratory for Mechanical Behavior of Materials, 12480 Xi’an Jiaotong University, Xi’an 710049, China; ⊥ Institute of Polymer Optoelectronic Materials and Devices, State Key Laboratory of Luminescent Materials and Devices, 26467 South China University of Technology, Guangzhou 510640, P. R. China; # College of New Materials and New Energies, 507738 Shenzhen Technology University, Shenzhen 518118, China; ¶ Beijing Key Laboratory of Ionic Liquids Clean Process, CAS Key Laboratory of Green Process and Engineering, State Key Laboratory of Multiphase Complex System, Institute of Process Engineering, 74526 Chinese Academy of Sciences, No. 1 Zhongguancun North Second Street, Haidian District, Beijing 100190, China; ∇ 57815 National Synchrotron Radiation Research Center, Hsinchu Science Park, Hsinchu 30076, Taiwan; ○ Department of Chemical Engineering, National Tsing Hua University, Hsinchu 30013, Taiwan; ⧫ Institute of Functional Nano & Soft Materials (FUNSOM), 12582 Jiangsu Key Laboratory of Advanced Negative Carbon Technologies Soochow University, Suzhou, Jiangsu 215123, P. R. China

**Keywords:** grazing-incidence small-angle neutron scattering, targeted deuteration, short-range aggregation, thick-film organic solar cells, ternary strategy, carrier transport connectivity

## Abstract

High-performance organic solar cells (OSCs) suffer from low active layer thickness tolerance, which is incompatible with large-scale printing technology originally envisioned for low-cost module manufacturing. Herein, by incorporating a large amount of small-molecule donor BTR-Cl into the prototypical PM6/Y6 blend film, we fabricated efficient ternary devices with a photoconversion efficiency of 17.7% at an active layer thickness of 300 nm, among the best-performing thick-film devices reported so far. To elucidate its morphological origin, we deuterated both Y6 and BTR-Cl to resolve their morphology in ternary blend films separately via grazing-incidence small-angle neutron scattering (GISANS). We observed enhanced short-range aggregation of both Y6 and BTR-Cl within the intermixed domains of the ternary blend film induced by the accelerated molecular assembly process. Those aggregates act as effective bridges between crystalline domains to improve connectivity in both donor and acceptor phases, resulting in enhanced carrier mobility, suppressed space charge accumulation, and consequently significantly improved thickness tolerance in ternary devices. Our work demonstrates the effectiveness of combined targeted deuteration and GISANS to resolve the complicated structures within multicomponent OSC active layers and highlights the critical role of amorphous nanomorphology in carrier transport connectivity and, consequently, the thickness tolerance of high-performance OSCs.

## Graphical abstract

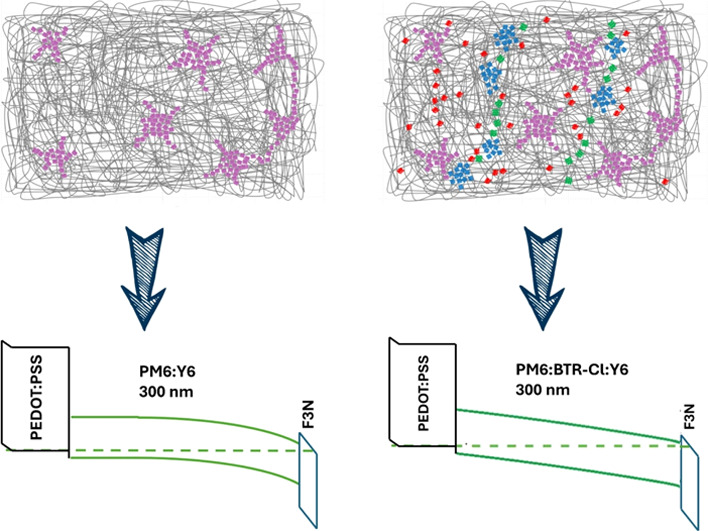

## Introduction

The record power conversion efficiency (PCE) of organic solar cells (OSCs) has recently surpassed 20%,^1^ approaching levels demanded for commercialization.^2–7^ However, most high-efficiency OSCs are fabricated with an optimal active layer thickness of around 100 nm, as further increasing the thickness results in a significant drop in the fill factor (FF) due to inefficient charge extraction (CE).^8–10^ Such a low thickness tolerance poses significant challenges for scaling up OSC manufacturing, as industrial-scale deposition of defect-free thin films typically demands a film thickness of over 300 nm.^11–14^ The restricted active layer thickness of OSCs also results in incomplete light absorption in thin-film devices that compromises the short-circuit current (*J*_SC_).^15^ Therefore, developing efficient thick-film devices is crucial to further improve the performance and scalability of OSCs, making it an essential step toward commercialization.

The low thickness tolerance of the OSC can be attributed to the inherently low carrier mobility of most organic semiconductors. Increasing the active layer thickness not only increases the distance that charge carriers must travel before getting collected but also reduces the carrier velocity by lowering the built-in field.^16–23^ The situation gets worse when the transport of electrons and holes becomes imbalanced, as the accumulation of slow charge carriers will lead to the formation of a space-charge region that further screens the built-in field, a phenomenon known as the space-charge-limited photocurrent (SCLPC).^18,24,25^ The microscopic origin of SCLPC has been extensively studied in fullerene-based OSCs, with proposed reasons including unintentional doping,^26^ energetic disorder,^19^ and unbalanced mobility.^27^ In contrast, a systematic study on the morphological origin of SCLPC in high-performance nonfullerene acceptor (NFA) OSCs is still lacking.

The most widely adopted approach to enhance the performance of thick-film OSCs is by adding a third component to the OSC active layers.^28–34^ In particular, small-molecule donors including DCRN5T and BTR derivatives have been incorporated into both fullerene- and NFA-based systems to fabricate efficient thick-film OSCs.^12,35–38^ While enhanced crystallinity is often proposed as the reason behind the improved performance, this correlation is not universally valid, as an increased crystallinity does not always translate to better charge extraction in blend films containing both crystalline and amorphous domains.^39,40^ The ternary strategy also poses challenges to morphology characterization, especially for NFA OSCs, as all three components share very similar molecular structures, making it almost impossible to resolve their individual morphologies within the blend films. As a result, the exact function of the third component in ternary OSCs remains elusive, which hinders the further development of efficient thick-film OSCs.

In this work, we introduced the small-molecule donor BTR-Cl as a third component into the prototypical PM6/Y6 system and achieved a champion PCE of 17.7% in devices with an active layer thickness of 300 nm. Detailed device analysis reveals that performance enhancement originates from enhanced and more balanced charge carrier mobilities, which suppress space-charge accumulation and improve charge extraction. To understand the morphological benefit of the ternary strategy, we deuterated both BTR-Cl and Y6 to enhance their scattering length density (SLD) under the neutron beam, allowing us to separately resolve their morphologies in blend films using grazing-incidence small-angle neutron scattering (GISANS). The short-range aggregation of d-Y6 within the intermixed domains, which we have previously identified to enhance interacceptor domain connectivity in thin PM6/d-Y6 film, becomes absent in the thick film, which explains its inferior electron transport. Encouragingly, d-Y6 aggregates reappear in the ternary blend film (PM6/d-Y6/BTRCl) regardless of the film thickness, suggesting that the incorporation of BTR-Cl promotes the aggregation of d-Y6 that leads to improved electron percolation. Additionally, the GISANS linecut of the PM6/d-BTR-Cl/Y6 blend film reveals a new scattering feature attributed to the short-range aggregation of d-BTR-Cl molecules. The simultaneously improved connectivity of both donor and acceptor phases accounts for the enhanced and more balanced charge extraction in ternary devices that leads to improved thickness tolerance. In situ absorption spectroscopy reveals a significantly accelerated assembly process of both donor and acceptor phases during the spin coating of thick ternary films, which explains the enhanced short-range aggregation. Overall, our work established a robust correlation between the thickness tolerance of NFA OSC and its amorphous nanomorphology, highlighting the critical role of short-range aggregations in assisting charge carrier percolation in thick-film devices. Those valuable insights will be applied to develop efficient thick-film OSCs that are compatible with industrial-scale manufacturing.

## Results and Discussion

### Device Characteristics

We first fabricated devices based on the blend film of PM6 and Y6, with or without the incorporation of BTR-Cl. Two active layer thicknesses were studied, which were 100 and 300 nm, respectively (corresponding to thin- and thick-film devices). The chemical structures and energy level diagram of the photoactive materials as well as the UV–vis absorption spectra of pure and blended films are presented in Figure S1. The complementary absorption spectra and cascaded energy levels between the donors and acceptors ensure efficient photon harvesting and charge generation. Devices were fabricated with a conventional structure of ITO/PEDOT:PSS/active layer/PNDIT-F3N/Ag (see details of device fabrication in the Supporting Information, with the statistics of device metrics shown in Table 1 and thickness measurements in Figure S3). As shown in Figure 1a, the binary thin-film device achieved a maximum power conversion efficiency (PCE) of 17.6%, with a short-circuit current density (*J*_SC_) of 27.4 mA cm^–2^, an open-circuit voltage (*V*_OC_) of 0.836 V, and a fill factor (FF) of 76.7%, consistent with previous results.^41,42^ Increasing the active layer thickness to 300 nm led to substantial performance degradation, with the PCE dropping to 11.4% (*J*_SC_ = 25.5 mA cm^–2^, *V*_OC_ = 0.802 V, and FF = 55.8%). In stark contrast, for ternary devices with an optimized PM6/BTR-Cl/Y6 weight ratio of 0.6:0.8:1.2, decent device performance was achieved in both thin- and thick-film devices. In particular, the ternary thick-film device achieved a PCE of 17.7%, with a *V*_OC_ of 0.831 V, a *J*_SC_ of 30.7 mA cm^–2^, and a FF of 69.2%, marking one of the highest PCEs reported for 300 nm-thick devices.^38,43,44^ We further increased the film thickness to 400 nm, and the ternary device still maintained a PCE of 15.9%, which is significantly higher than the 10.0% achieved by the binary device (Figure S4 and Table S1). The results of external quantum efficiency (EQE) measurements (Figure 1b) further support these findings, with the calculated *J*_SC_ obtained from the integration of EQE spectra matching well with the measured *J*_SC_. We further studied the variation of the PCE retention rate (the PCE ratio between thick- and thin-film devices) with the weight ratio of BTR-Cl incorporated. As shown in Table 1 and Figure S5, the PCE retention rate increases monotonically from 64.8% to 95.2% as the BTR-Cl content rises from 0% to 66.7%, followed by a rapid decline upon further increasing the BTR-Cl content. This highlights the critical role of the optimized ternary mixing morphology for improving the thickness tolerance of OSCs, as discussed in detail below. We also fabricated BTR-Cl:Y6 devices with 100 and 300 nm active layers (Figure S6). The 300 nm device showed a PCE of 12.2%, higher than that of the PM6/Y6 device with the same thickness, indicating the advantage of BTR-Cl in thick-film device fabrication.

**Table 1:** Performance of the Optimized OSCs under Illumination of AM 1.5 G, 100 mW cm^–2^ ^d^

Additive	*V*_OC_ (V)	PCE_d_ (%)	Fill Factor (%)	*J*_SC_ (mA/cm^2^)	*J*_SC_ cal (mA/cm^2^)
Binary100 nm	0.836 (0.836 ± 0.002)	17.6 (17.4 ± 0.3)	76.7 (76.8 ± 1.0)	27.4 (27.3 ± 0.2)	26.21
Binary 300 nm	0.802 (0.800 ± 0.003)	11.4 (11.2 ± 0.1)	55.8 (55.8 ± 1.1)	25.5 (25.0 ± 0.2)	25.97
Ternary 100 nm^a^	0.832 (0.835 ± 0.002)	17.7 (17.6 ± 0.1)	76.6 (76.2 ± 0.8)	27.7 (27.3 ± 0.6)	26.61
Ternary 300 nm^a^	0.816 (0.813 ± 0.003)	15.5 (15.0 ± 0.3)	64.1 (63.0 ± 1.6)	29.5 (29.3 ± 0.5)	28.26
Ternary 100 nm^b^	0.840 (0.837 ± 0.002)	18.6 (18.2 ± 0.2)	77.9 (77.6 ± 0.2)	28.3 (28.0 ± 0.2)	26.70
Ternary 300 nm^b^	0.831 (0.825 ± 0.010)	17.7 (17.3 ± 0.8)	69.2 (69.4 ± 3.9)	30.7 (30.3 ± 0.3)	29.17
Ternary 100 nm^c^	0.834 (0.831 ± 0.004)	17.5 (17.2 ± 0.2)	74.9 (74.7 ± 0.4)	28.1 (27.7 ± 0.4)	26.40
Ternary 300 nm^c^	0.814 (0.807 ± 0.005)	13.2 (12.2 ± 0.7)	56.1 (52.2 ± 2.8)	28.8 (29.1 ± 0.4)	28.39

a Weight ratio for PM6/BTR-Cl/Y6 is 0.8:0.4:1.2.

b Weight ratio for PM6/BTR-Cl/Y6 is 0.6:0.8:1.2.

c Weight ratio for PM6/BTR-Cl/Y6 is 0.4:1.2:1.2.

d Data obtained from eight devices.

**Figure 1:**
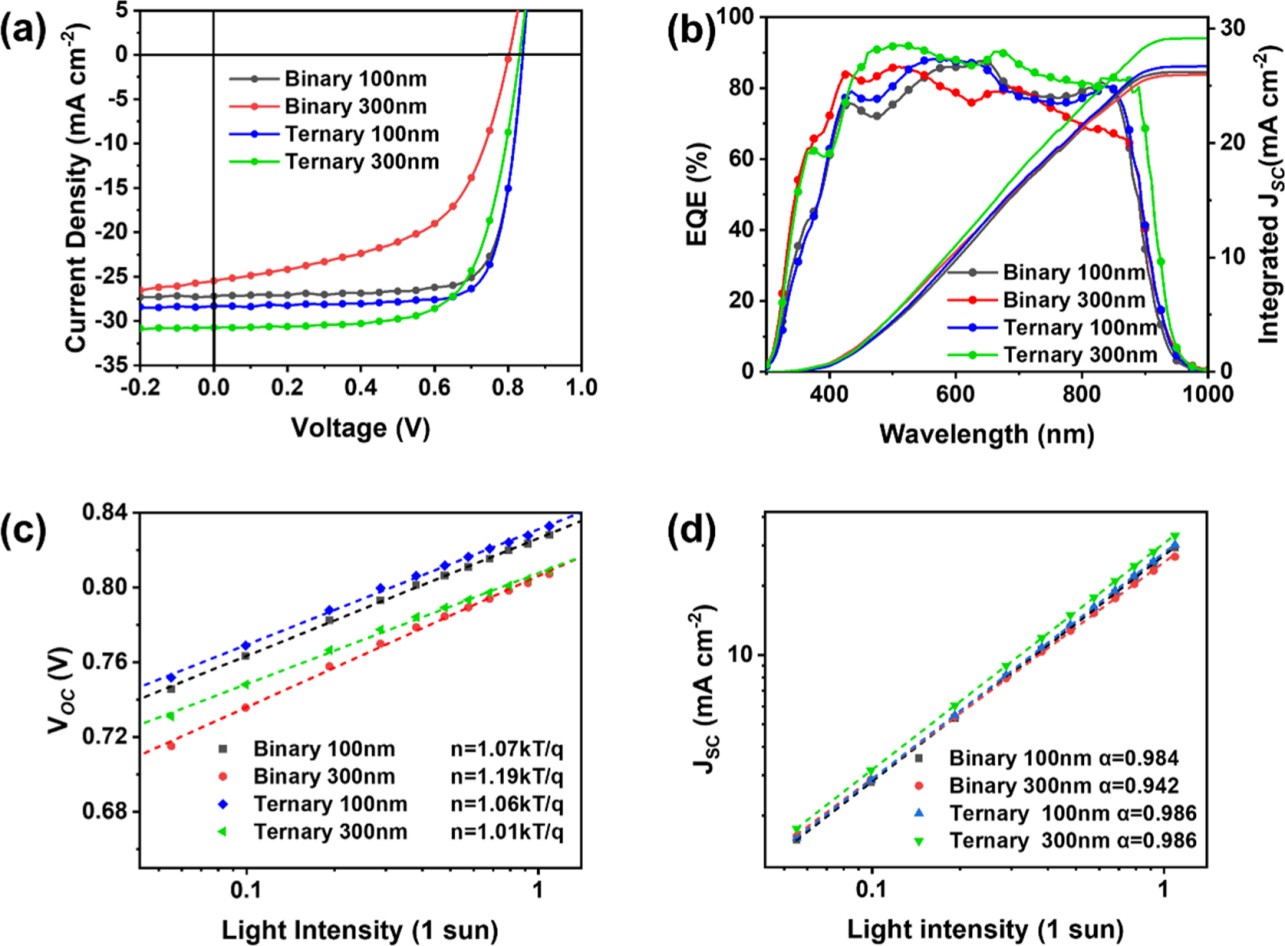
(a) The best-performing *J*–*V* curves; (b) EQE response and integrated *J*_SC_ of best-performing devices; (c,d) *V*_OC_ and *J*_SC_ versus light intensity for the OSC devices treated with different conditions.

Carrier recombination mechanisms were investigated through *J*_SC_ and *V*_OC_ dependence on light intensity (*P*_light_).^45,46^ The slope values of *V*_OC_–ln (*P*_light_) curves (Figure 1c) for 100 nm binary and ternary devices are very close to unity (1.07 *k*_B_ *T*/*q* and 1.06 *k*_B_ *T*/*q*, where *k*_B_ is the Boltzmann constant, *T* is the temperature, and *q* is the elementary charge), indicating suppressed trap-assisted recombination and dominant bimolecular recombination. However, binary 300 nm devices exhibit an increased trap-assisted recombination (1.19 *k*_B_ *T*/*q*), while ternary 300 nm devices successfully suppress this pathway (1.01 *k*_B_ *T*/*q*).^47^ Similarly, the *J*_SC_ ∝ *P*_light_^α^ analysis gives rise to an α exponent of 0.984, 0.942, 0.986, and 0.986 for binary 100 nm, binary 300 nm, ternary 100 nm, and ternary 300 nm devices, respectively. The ternary devices effectively reduce bimolecular recombination, particularly in 300 nm devices, as indicated by higher α values (Figure 1d). The overall suppressed recombination in ternary 300 nm devices is attributed to improved film morphology, which will be discussed later.

To understand the origin of performance enhancement in the ternary thick-film device, we performed photocurrent (*J*_ph_) measurements within the light intensity (*P*_light_) range of 0.57–1.59 sun. Under each light intensity, *J*_ph_, defined as the difference between the dark and light current, is plotted against the effective voltage (*V*_eff_), which is obtained by subtracting the external applied voltage from the voltage at zero photocurrent density. As shown in Figure S7, two stages can be identified in the *J*_ph_–*V*_eff_ curves of the thin-film devices. This includes a linear region (region I) at small *V*_eff_ due to the recombination between photogenerated and injected charge carriers, followed by a saturation region at intermediate and large *V*_eff_ (region II). As shown in Figures 2a and 2b, an additional region appears in the binary thick-film device at intermediate *V*_eff_ (region III), where *J*_ph_ scales linearly with $\sqrt{V_{eff}}$ . With increasing *P*_light_, the width of region III extends toward a higher *V*_eff_, and the magnitude of *J*_ph_ at a given *V*_eff_ scales with *P*_light_^0.78^. This indicates the presence of space-charge-limited photocurrent (SCLPC) in binary thick-film devices (see detailed derivations in Supplementary Note 1).^27^ SCLPC arises from imbalanced charge extraction; namely, one type of charge carrier is extracted at a slower rate than the other, so the slower charge carriers pile up near the extracting electrode, screening the built-in field that further degrades charge extraction. On the other hand, the SCLPC region disappears in the ternary thick-film device (Figure 2d,e), suggesting that the incorporation of BTR-Cl leads to a much-enhanced and more balanced extraction in thick-film devices.

**Figure 2:**
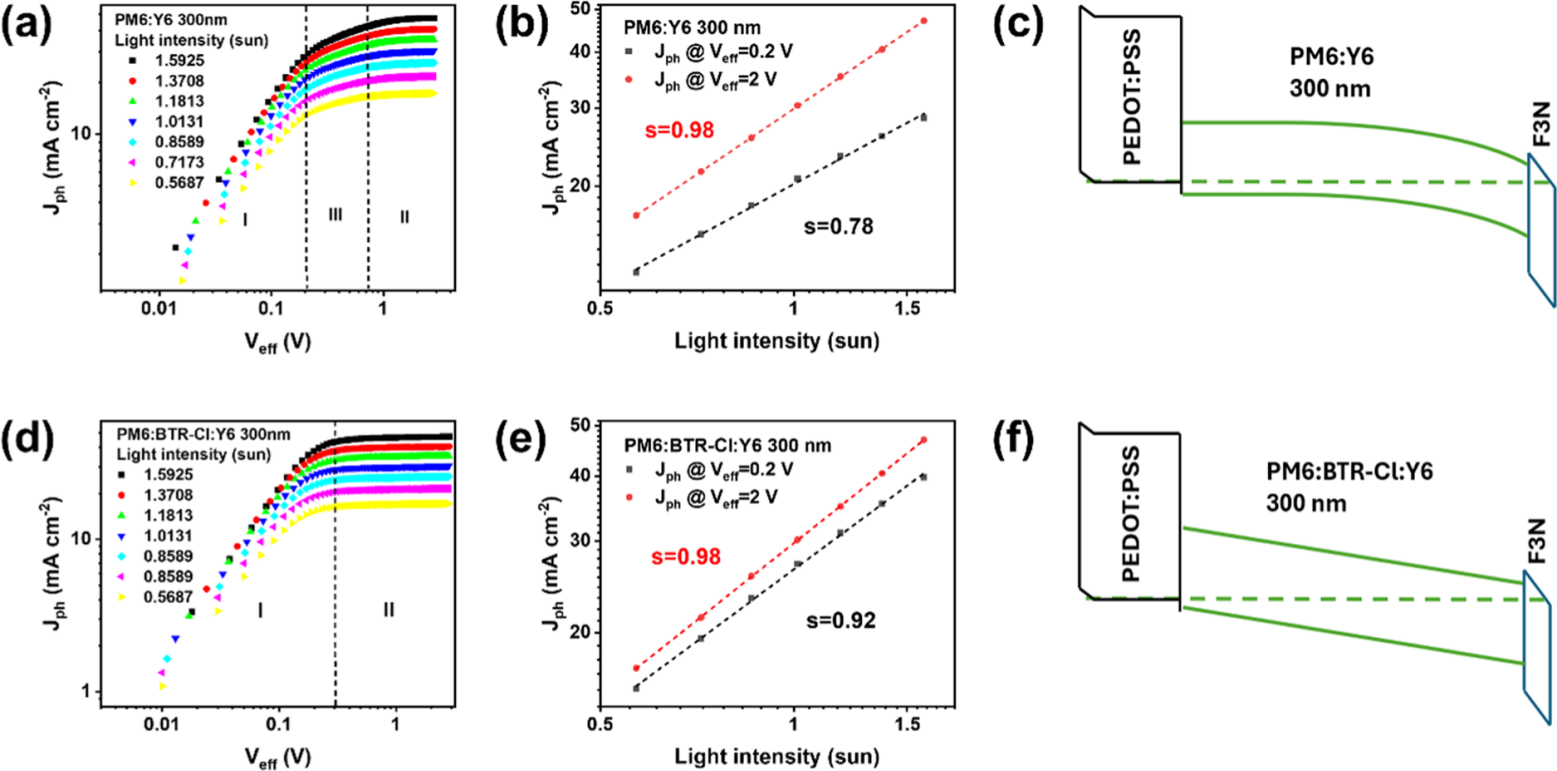
(a,d) Incident light power dependence of the photocurrent versus the effective voltage; (b,e) incident light power dependence of the photocurrent extracted at *V*_eff_ = 0.2 and 2 V; (c,f) energy band diagrams of binary and ternary thick devices.

To examine the location of the space-charge-limited region, we performed film-depth-dependent light absorption spectroscopy measurements to obtain the depth- and wavelength-dependent photogeneration profiles.^48^ As shown in Figure S8, while the photogeneration profile appears uniform in thin-film devices, it becomes concentrated near the anode, where the incident photons first arrive in thick-film devices. While holes can be easily extracted by the anode, electrons need to travel a much longer distance, so they accumulate and form a space charge region near the cathode, leaving a large neutral region in the rest of the active layer that compromises charge extraction, as depicted in Figure 2c. In contrast, ternary thick-film devices remain fully depleted under illumination, which allows efficient charge extraction throughout the entire active layer, as shown in Figure 2f.

In addition to nonuniform photogeneration, the formation of the space-charge-limited region can also be affected by unintentional doping,^26^ energetic disorder,^19^ and unbalanced mobility.^27^ As shown in Figure S9, similar capacitance–voltage curves were obtained for both binary and ternary thick-film devices, ruling out the potential impact of unintentional doping on thick-film device performances.^49^ Next, we carried out charge extraction measurements to obtain the variation of the charge carrier density (*n*) as a function of *V*_OC_. This allows us to determine the Urbach energy (*E*_ch_) from the slope (γ) of the semilog plot via the relation *E*_ch_ = 1/2γ.^19,50^ As shown in Figure S10–S12, the *E*_ch_ values for all studied devices are around 32 meV, close to the thermal energy (∼25 meV) at room temperature. This confirms that shallow trap density does not play a decisive role in thick-film device performance.^51^ Additionally, the ternary thick film shows a faster photocurrent decay compared to the binary counterpart (Figure S11), which further indicates the improved charge extraction in ternary films.

To evaluate the electron and hole mobility, we performed space-charge-limited-current (SCLC) measurements on single-carrier devices.^52^ Results summarized in Table S2 and Figure S13 show that binary devices suffer from unbalanced electron mobilities, resulting in unbalanced μ_h_/μ_e_ ratios of 1.35 (100 nm) and 2.33 (300 nm). In contrast, ternary devices show much improved electron transport and more balanced ratios of 0.99 (100 nm) and 1.09 (300 nm). Effective mobility ( $\mu_{eff}=\frac{{2\mu }_{e}\mu_{h}}{\mu_{e}+\mu_{h}}$ ) at short-circuit conditions derived from voltage-dependent capacitance spectroscopy (Figure S14) also consistently shows marked improvement in ternary devices, increasing from 3.45 × 10^–4^ cm^2^ V^–1^ s^–1^ to 5.45 × 10^–4^ cm^2^ V^–1^ s^–1^ in thin-film devices and 2.41 × 10^–4^ cm^2^ V^–1^ s^–1^ to 5.98 × 10^–4^ cm^2^ V^–1^ s^–1^ in thick-film devices. Therefore, it can be concluded that both the nonuniform photogeneration profile and unbalanced mobility (inferior electron transport) contribute to the formation of SCLPC, which deteriorates the performance of the binary thick-film device. In contrast, the much enhanced and more balanced carrier mobility in the ternary thick-film device counterbalances the undesirable effect of the nonuniform photogeneration profile, resulting in fully depleted active layers that enhance charge extraction.

### Ternary Morphology Revealed by GISANS and Deuteration

To understand the morphological origin of the enhanced thickness tolerance, it is essential to comprehensively characterize the ternary morphology. Our previous work^53^ demonstrated that selective deuteration of NFA molecules can substantially enhance their scattering length densities (SLDs) under the neutron beam, with negligible impact on their optoelectronic properties. The enhanced scattering contrast enables the quantitative determination of both crystalline and amorphous domain sizes of the deuterated component in blend films by using GISANS. Herein, we selectively deuterated both Y6 and BTR-Cl to leverage this advantage. While the synthesis route of deuterated Y6 (d-Y6) followed our previously established protocol,^53^ we developed a new synthetic route for deuterated BTR-Cl (d-BTR-Cl), as illustrated in Figure 3a. To simplify the synthesis process and reduce costs, we limited deuteration to the alkyl side chains. The intermediate and final molecular structures of d-BTR-Cl are validated by NMR spectroscopy and mass spectrometry (Figures S15–S31). Atomic force microscopy (AFM) measurements (Figure 3b,c) revealed similar surface morphologies for BTR-Cl and d-BTR-Cl films, with root-mean-square roughness (*R*_q_) values of 2.235 and 2.000 nm, respectively. Ultraviolet–visible (UV–vis) absorption spectra (Figure 3d) also showed good consistency with 0–1 and 0–0 peaks appearing at 577/618 nm for BTR-Cl and 576/616 nm for d-BTR-Cl. We then determined the ionization energies (IEs) of d-BTR-Cl and BTR-Cl through ultraviolet photoelectron spectroscopy (UPS) (Figure S33), and the corresponding electron affinities (EAs) were estimated by adding the IEs with the optical gaps derived from UV–vis absorption spectra (Figure 3e). The calculated IE/EA values of BTR-Cl and d-BTR-Cl are 5.14/3.34 eV and 5.07/3.27 eV, respectively. Furthermore, OPV devices incorporating d-BTR-Cl exhibited nearly identical device performance compared to those with nondeuterated BTR-Cl (Figure S34). These results confirm that deuteration minimally impacts the morphology and optoelectronic properties of BTR-Cl, consistent with our previous conclusion on Y6.

**Figure 3:**
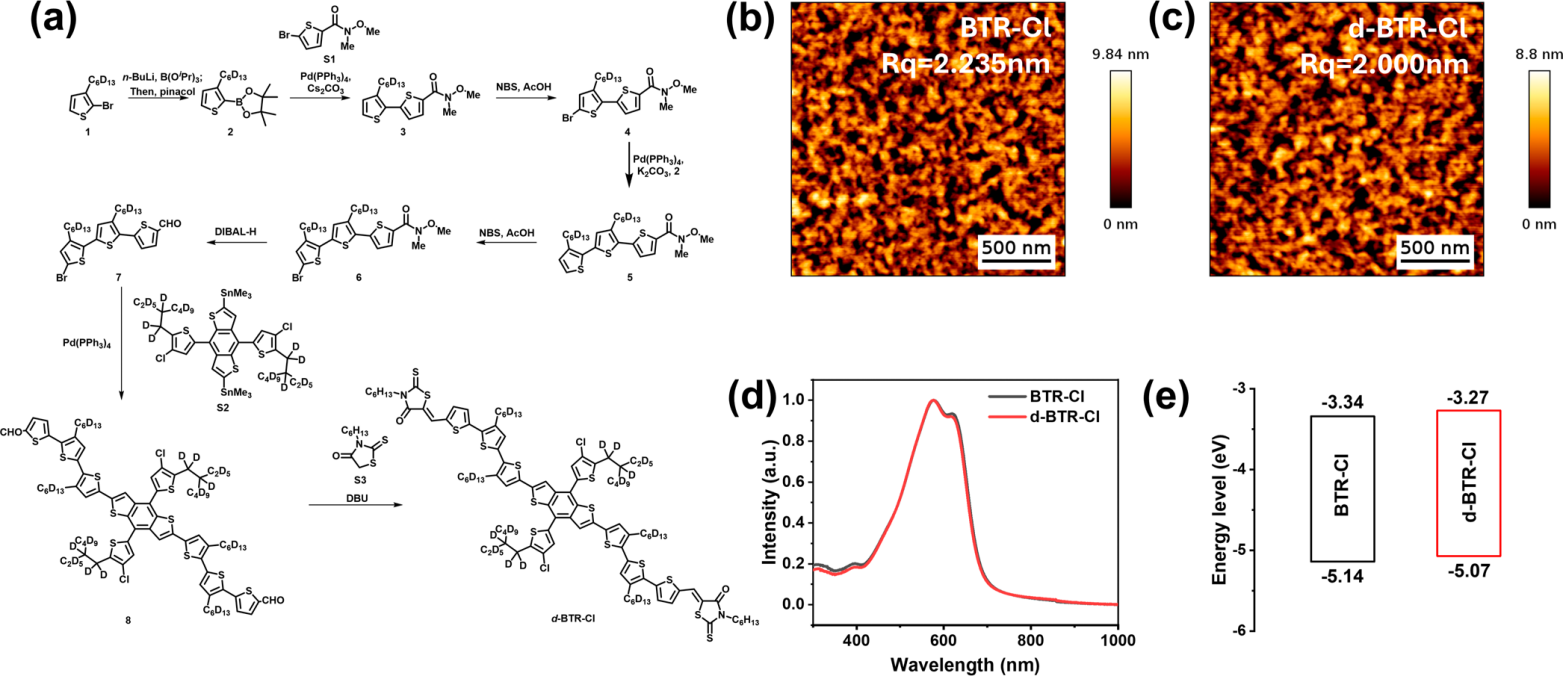
(a) Synthetic route and chemical structure of d-BTR-Cl; (b,c) AFM images, (d) UV absorption spectra, and (e) energy level diagrams of BTR-Cl and d-BTR-Cl.

Next, time-of-flight (TOF)-GISANS measurements were performed on the pure films of BTR-Cl and d-BTR-Cl to calculate their SLDs under neutron exposure. 2D GISANS patterns of pure BTR-Cl and d-BTR-Cl films, with the specular peak (*q*_S_) and the Yoneda peak (*q*_Y_) marked by the yellow and orange lines, are shown in Figure S35. The SLDs were obtained from the linear fitting of the calculated critical angle versus wavelengths from 4.5 to 8.5 Å. The SLDs of BTR-Cl and d-BTR-Cl are fitted to be (3.2 ± 0.2) × 10^–6^ Å^–2^ and (5.3 ± 0.2) × 10^–6^ Å^–2^, respectively. Notably, the SLD of d-BTR-Cl is higher than the SLD of Y6 ((2.3 ± 0.1) × 10^–6^ Å^–2^) and PM6 ((1.2 ± 0.1) × 10^–6^ Å^–2^), as reported in previous work,^53^ while the SLD of d-Y6 ((6.4 ± 0.4)×10^–6^ Å^–2^) is higher than those of BTR-Cl and PM6. The much higher SLDs of d-BTR-Cl and d-Y6 allow their morphology to be highlighted during the GISANS measurements of binary and ternary blend films.

Figures 4 and S36 show 2D GISANS patterns and in-plane intensity profiles of binary and ternary blended films with different thicknesses. The intensity profiles were fitted with the product of a spherical form factor and a fractal/hard-sphere structure factor to extract the size of crystalline/amorphous domains.^54–58^ The fitting details can be found in Supplementary Note 2, and the fitting results are summarized in Table S3. In the PM6/d-Y6 binary thin film, we obtained a crystalline d-Y6 domain size of 16.7 nm and an amorphous d-Y6 aggregate size of 8.7 nm, consistent with our previous results. However, the scattering feature corresponding to the amorphous d-Y6 aggregates disappears in the binary thick film. In our previous works,^53,59^ we have identified electron trapping in isolated NFA domains as a major loss mechanism in high-performance OSCs due to the large LUMO offset between donors and NFAs. The presence of short-range d-Y6 aggregates is expected to significantly improve electron percolation within the intermixed domains, which suppresses electron trapping and enhances the device performance. Therefore, we propose that the disappearance of short-range d-Y6 aggregates in the binary thick film should account for the inferior electron transport and the SCLPC observed in the binary thick-film device. Remarkably, the incorporation of BTR-Cl restored the amorphous d-Y6 aggregates in the ternary (PM6/BTR-Cl/d-Y6) thick film with a crystalline d-Y6 domain size of 26.6 nm and an amorphous d-Y6 domain size of 9.1 nm. Additionally, when d-BTR-Cl was highlighted in the ternary thick film (PM6/d-BTR-Cl/Y6), two scattering features can be identified and assigned to the crystalline and amorphous d-BTR-Cl domains with sizes of 16.8 and 9.7 nm, respectively. Those results suggest that BTR-Cl not only forms short-range aggregates within the intermixed domains but also promotes the aggregation of Y6, as depicted in the schematics in Figures 4c, 4f, and 4i. Consistently, a notable red-shift of the Y6 absorption peak by around 15 nm in the blend film was also observed upon incorporating BTR-Cl, further confirming that BTR-Cl promotes Y6 aggregation in ternary blend films. Overall, the simultaneously enhanced interdomain connectivity of both donor and acceptor phases leads to more balanced charge extraction, resulting in improved thickness tolerance observed in ternary devices.

**Figure 4:**
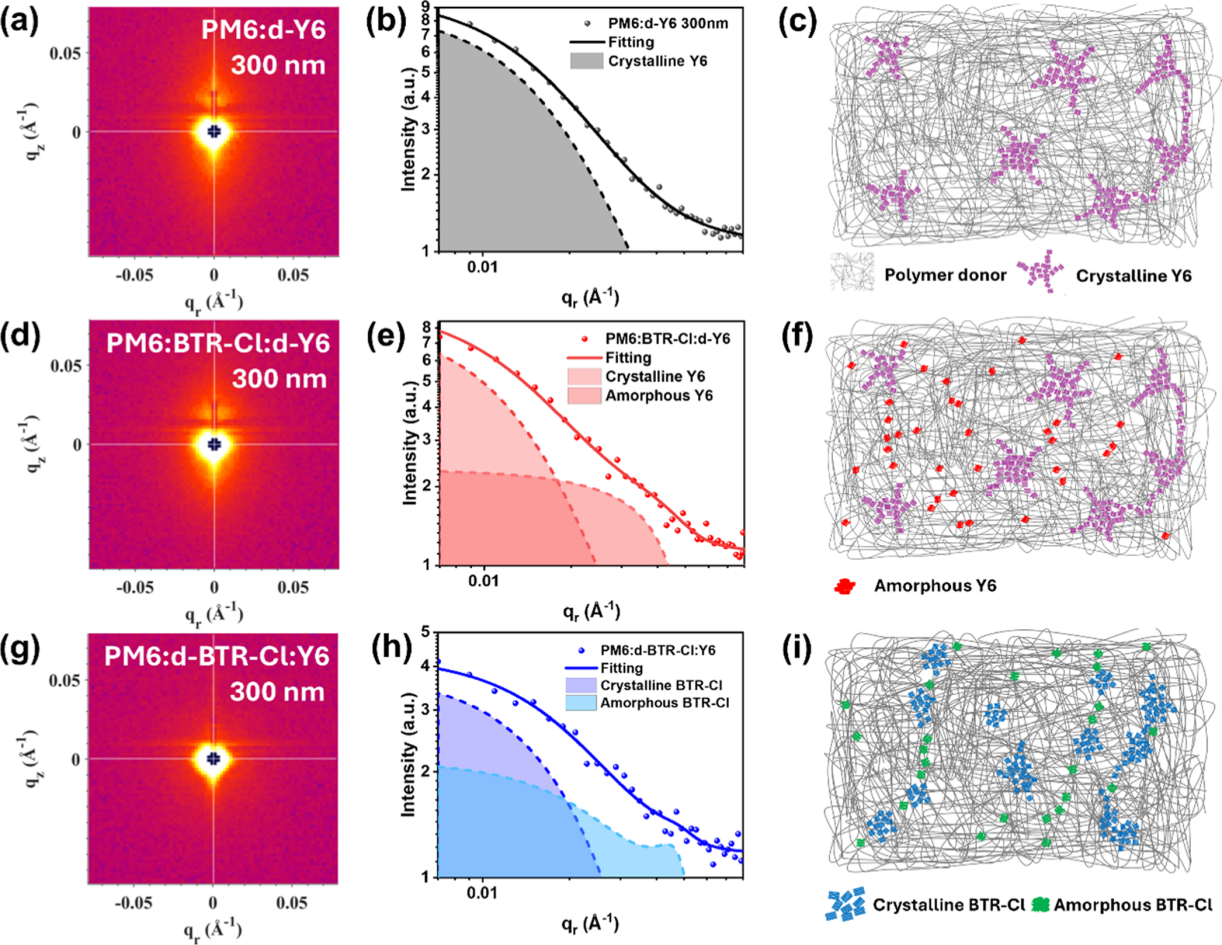
TOF-GISANS measurements of 300 nm blend films. (a,d,g) TOF-GISANS patterns of PM6/d-Y6, PM6/BTR-Cl/d-Y6, and PM6/d-BTR-Cl/Y6 with their horizontal linecuts (dots) with best fits (solid lines) shown in (b), (e), and (h), respectively. (c,f,i) The schematics show the main features that give rise to scattering contrasts in GISANS measurements.

### Molecule Assembly Kinetics and Other Morphology Results

To explore the origin of enhanced short-range aggregation, we examined the film formation kinetics during spin coating using in situ UV–vis measurements.^60,61^ The raw data are listed in Figure S34, and the 2D contour maps are shown in Figure 5a–d. The evolution of peak locations of the donor and acceptor is shown in Figure 5e,f. In general, thick films exhibit prolonged transition duration from solution to film state owing to a lower spin speed. At the same film thickness, the donor assembly kinetics are similar for binary and ternary films (Figure 5e), yet the acceptor assembly kinetics become noticeably different (Figure 5f). Upon incorporating BTR-Cl, the solution to film transition time decreases from 1.9 to 1.5 s for thin films and from 6.2 to 4.8 s for thick films, respectively. The long transition time in the binary thick film allows crystalline domains to grow at the expense of short-range aggregates, similar to the mechanism of Ostwald ripening.^62,63^ This is consistent with our previous results showing that the high-boiling-point solvent results in smaller amorphous aggregates compared to low-boiling-point solvents.^53^ Therefore, the accelerated molecular assembly process in ternary films allows more short-range Y6 aggregates to be retained within the intermixed domains, resulting in much-improved interdomain connectivity.

**Figure 5:**
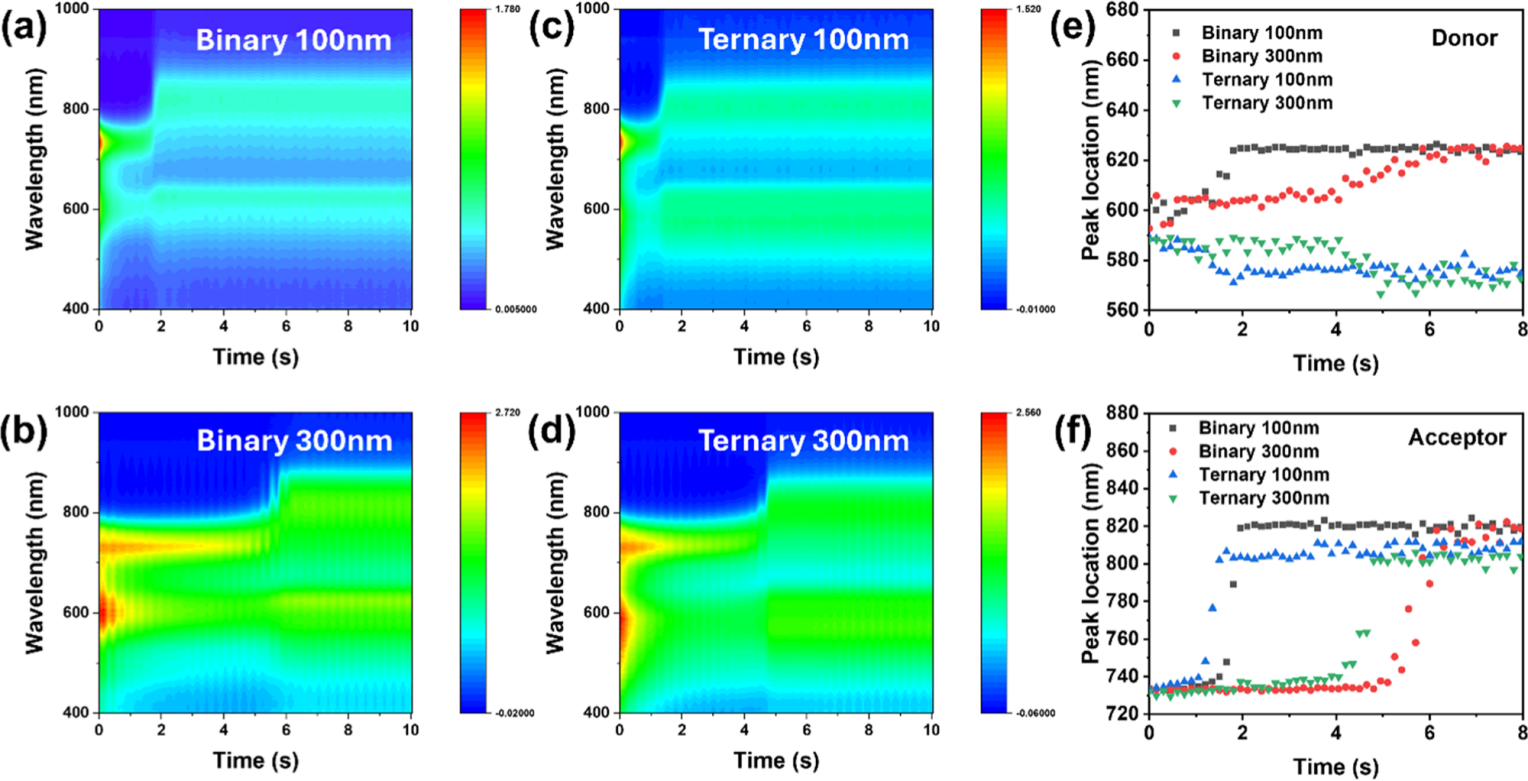
(a–d) Time-dependent contour maps of UV–vis absorption spectra; (e,f) extracted peak locations of the donor and acceptor with binary and ternary blends.

In addition, tapping-mode AFM was performed to examine the surface morphologies of blended films. The topography and phase images (Figures 6a,b, S35, and S36) revealed an increase in the root-mean-square (RMS) roughness of the active layer with the addition of BTR-Cl. Conductive-AFM (c-AFM) was utilized to further probe the conductive pathway along the vertical direction. By leveraging the highest occupied molecular orbital (HOMO) offset between donor and acceptor materials, regions of low luminescence with elevated local hole currents can be identified as donor-rich domains.^64^ As shown in Figure 6c,d and Table S4, the average current value is 215.5 pA and 314.9 pA for binary and ternary 300 nm films, respectively, indicating more conductive pathways formed in the ternary system, with the presence of amorphous aggregates of both Y6 and BTR-Cl.^65^ However, there are notable differences in the spatial variations of local hole currents, quantified as the RMS currents between different films. Specifically, the RMS current of the ternary 300 nm film (59.13 pA) is larger than the binary 300 nm film (43.90 pA), suggesting that the extent of phase separation between donor and acceptor is higher in the ternary system, consistent with GISANS results, and responsible for the lower biomolecule recombination loss. GIWAXS measurements were conducted to acquire information about the molecule packing behaviors of the film (Figures 6e–i and S40,S41). The overall scattering intensity of thick films is significantly higher than that of thin films as expected due to the increased interaction and scattering of X-rays with a larger volume of materials. The π–π peak positions are all located at q_*z*_ of around 1.80 Å^–1^ (*d* = 3.50 Å), a signature of face-on-oriented packing, while the crystallite coherence lengths (CCLs) of the ternary blend film are relatively larger (Table S5), consistent with the crystalline domain variation from GISANS results. It is important to note that the crystalline domain size extracted from the GISANS fitting refers to the correlation length of periodic nanostructures, which can include multiple coherent crystalline grains. This is typically larger than the crystallite size or CCL derived from GIWAXS, which reflects the size of individual ordered regions.^53^

**Figure 6:**
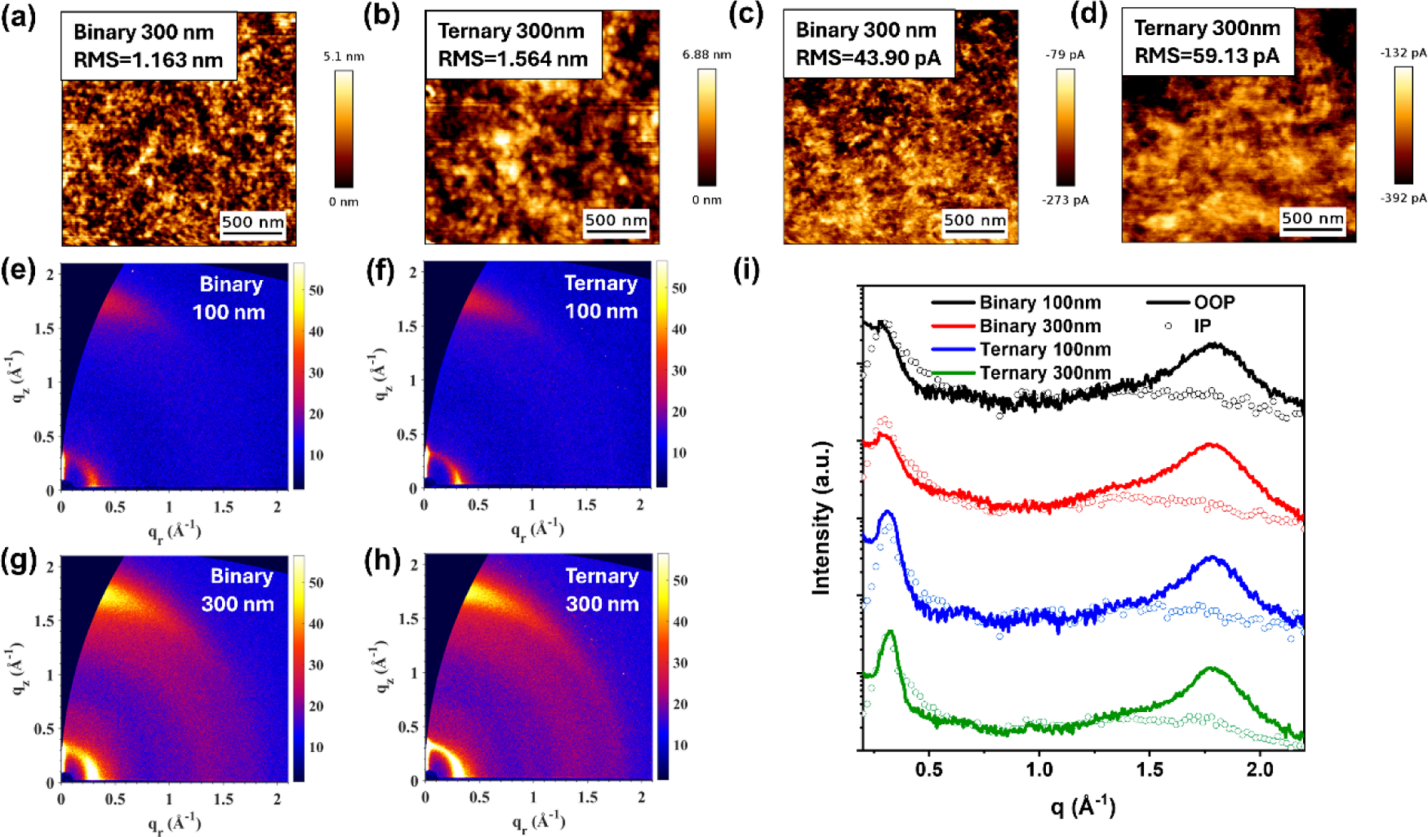
(a–d) AFM topography images and c-AFM images for binary and ternary thick films; (e–h) GIWAXS 2D plots for binary and ternary films with different thicknesses; and (i) corresponding linecut.

### Conclusion

In conclusion, we fabricated high-performance organic solar cells (OSCs) with excellent thickness tolerance based on PM6, BTR-Cl, and Y6. The optimized 300 nm ternary device achieved a power conversion efficiency (PCE) of 17.7%, one of the highest reported for OSCs of this thickness. The morphological origin of the performance enhancement is revealed through a combination of targeted deuteration and GISANS. Our results indicate that BTR-Cl not only forms short-range aggregates within the intermixed domains but also facilitates the short-range aggregation of Y6. Benefiting from the simultaneously enhanced connectivity in both donor and acceptor phases, ternary devices exhibit much enhanced and more balanced charge carrier mobilities, which suppress space charge accumulation in thick-film devices and result in excellent thickness tolerance. We further demonstrated that the formation of amorphous clusters is linked to the molecular assembly process during spin coating, with a shorter assembly time favoring the formation of shorter-range aggregates. Our work provides clear guidelines to improve the thickness tolerance of OSCs by engineering the amorphous nanomorphology within the active layer, paving the way toward efficient and scalable OSCs.

## Experimental Section

### Materials

Unless stated otherwise, all solvents and reagents were received from commercial sources and used without further purification. Chloroform (99.5%) and 1,4-diiodobenzene (DIB) (99.0%) were purchased from Sigma-Aldrich. PM6, BTR-Cl, and Y6 were purchased from Solarmer Inc. (Beijing). PNDIT-F3N was purchased from OptiFocus Ltd. Ag was purchased from ZhongNuo Advanced Material (Beijing) Technology Co., Ltd. PEDOT:PSS was purchased from Shanghai VIZU Chemical Technology Co., Ltd.

### Characterizations of Deuterated Materials

All reactions were carried out under an argon atmosphere with dry solvents under anhydrous conditions unless otherwise noted. Reagents were purchased at the highest commercial quality and used without further purification unless otherwise stated. Deuterated Y6 was synthesized according to our previous literature.^53^ Compound 1 and S2 were prepared according to the known literature methods, using deuterated compounds 1-bromohexane-D13 and 1-bromo-2-ethylhexane-D17 as starting materials, respectively.^66,67^ Reactions were monitored by thin layer chromatography (TLC) carried out on MilliporeSigma glass TLC plates (silica gel 60 coated with *F*_254_, 250 μm) using UV light for visualization. SiliaFlash P60 silica gel (particle size: 40–63 μm, pore size: 60 Å) was used for flash column chromatography. NMR spectra were recorded on a Bruker AVANCE III 400 MHz or a Bruker Avance III HD 600 MHz NMR spectrometer. The spectra were calibrated by using residual undeuterated solvents (for ^1^H NMR) and deuterated solvents (for ^13^C NMR) as internal references: undeuterated chloroform (δ_H_ = 7.26 ppm) and CDCl_3_ (δ_C_ = 77.16 ppm). The following abbreviations are used to designate multiplicities: s = singlet, d = doublet, and m = multiple. High-resolution mass spectra (HRMS) were recorded on a Thermo Scientific Q- Exactive mass spectrometer.

### Device Fabrication and Characterization

All the devices are based on a conventional sandwich structure, patterned ITO glass/PEDOT:PSS/active layer/PNDIT-F3N/Ag. The ITO substrates were first scrubbed by detergent and then sonicated with deionized water, acetone, and isopropanol subsequently. The glass substrates were treated by UV–ozone for 20 min before use. PEDOT:PSS (Heraeus Clevios P VP AI 4083) was spin-cast onto the ITO substrates at 4000 rpm for 30 s and then dried at 120 °C for 20 min in air. To obtain devices with different thicknesses, the active layers for the binary blends and ternary blends were spin-coated from different concentrations and at different spin-coating speeds. For binary blends with 100 nm thickness, the D/A ratio is 1.2:1, the total concentration is 15.4 mg/mL, and the spin-coat speed is 3000 rpm. For binary blends with 300 nm thickness, the D/A ratio is 1.2:1, the total concentration is 24 mg/mL, and the spin-coat speed is 1000 rpm. For ternary blends (PM6/BTR-Cl/Y6 = 0.8:0.4:1.2 in weight ratio) with 100 nm thickness, the total concentration is 16.6 mg/mL, and the spin-coat speed is 3000 rpm. For ternary blends (PM6/BTR-Cl/Y6 = 0.8:0.4:1.2 in weight ratio) with 300 nm thickness, the total concentration is 26 mg/mL, and the spin-coat speed is 1000 rpm. For ternary blends (PM6/BTR-Cl/Y6 = 0.6:0.8:1.2 in weight ratio) with 100 nm thickness, the total concentration is 18.2 mg/mL, and the spin-coat speed is 3000 rpm. For ternary blends (PM6/BTR-Cl/Y6 = 0.6:0.8:1.2 in weight ratio) with 300 nm thickness, the total concentration is 28 mg/mL, and the spin-coat speed is 1000 rpm. For ternary blends (PM6/BTR-Cl/Y6 = 0.4:1.2:1.2 in weight ratio) with 100 nm thickness, the total concentration is 19.6 mg/mL, and the spin-coat speed is 3000 rpm. For ternary blends (PM6/BTR-Cl/Y6 = 0.4:1.2:1.2 in weight ratio) with 300 nm thickness, the total concentration is 29 mg/mL, and the spin-coat speed is 1000 rpm. The post-thermal annealing treatment is at 100 °C for 10 min. A thin PNDIT-F3N layer (∼5 nm) was coated on the active layer, followed by the deposition of the Ag electrode (100 nm).

The current density–voltage (*J*–*V*) curves of devices were measured using a Keithley 2400 Source Meter in a glovebox under AM 1.5G (100 mW cm^–2^) using an Enlitech solar simulator. The scan was performed in the forward direction with a step size of 0.01 V and a dwell time of 1 ms per point. The absorption spectra of thin films were measured using a PerkinElmer Lambda 950 UV/vis/IR on quartz substrates. The external quantum efficiency (EQE) was measured by a QE/IPCE system (Enli Technology Co. Ltd.) in the wavelength range 300–1000 nm. The active layer surface was characterized under ambient conditions via an atomic force microscope (Bruker, Dimension Icon) using a Tap300Al-G tip (40 N/m) for topography and phase characterizations. Measurements were carried out using the tapping mode with the tip oscillating at a fixed frequency (∼300 kHz) and amplitude above the sample surface. c-AFM measurements were conducted using an ElectriCont-G tip (0.2 N/m, coated with Pt/Ir) in the contact mode. A positive bias was applied to the sample substrate so that holes could be injected from PEDOT:PSS to the active layer before being collected by the tip, which was recorded as a negative current flow. Considering the relatively flat surfaces of all films studied, the current contrast in c-AFM mappings mainly arises from the HOMO offset between PM6 and Y6-rich domains, resulting in different injection barriers. The scan area and speed were 2.0 μm × 2.0 μm and 1 Hz. The root-mean-square (RMS) fluctuations in height and current were obtained by using the JPKSPM Data Processing software package. The GIWAXS measurements were carried out with a Xeuss 2.0 SAXS/WAXS laboratory beamline S3 using a Cu X-ray source (8.05 keV, 1.54 Å) and a Pilatus3R 300 K detector. The incidence angle is 0.2°. The GISAXS measurements were conducted at the 23A SWAXS beamline at the National Synchrotron Radiation Research Center, Hsinchu, Taiwan, using a 10 keV primary beam, a 0.15° incident angle, and Pilatus 1M-F. The GISANS measurements were conducted at the BL-01 (SANS) beamline at the China Spallation Neutron Source (CSNS). The sample-to-detector distance was 4 m, and the chosen wavelength range was 1.2 Å–9.5 Å. All samples were measured for 15 h to obtain sufficient statistics.

### Transient Photovoltage (TPV) and Charge Extraction (CE) Analyses

The TPV technique using PAIOS is based on monitoring the photovoltage decay upon a small optical perturbation during different constant bias light intensities (using the same white LED for TPC measurements and under open-circuit conditions). Variable bias light intensities lead to a range of *V*_OC_ to be studied. A small optical perturbation (<3% of the *V*_OC_, so that Δ*V*_OC_ ≪ *V*_OC_) is applied. The subsequent voltage decay is then recorded to directly monitor nongeminate charge carrier recombination. The photovoltage decay kinetics of all devices follows a monoexponential decay: δ*V* = *A* exp­(−*t*/τ), where *t* is the time and τ is the charge carrier lifetime.^68,69^

The CE technique was used to measure the charge carrier density under open-circuit voltage conditions. The device is illuminated and kept an open circuit. After the light is turned off, the voltage is set to zero or taken to a short-circuit condition within a few hundreds of nanoseconds to extract the charges. To obtain the number of extracted charges, the current is integrated. Using the charge carrier lifetime obtained from TPV and charge carrier density from CE, we plotted the charge carrier lifetimes and charge carrier densities. The charge carrier lifetime follows a power law relationship with charge density: τ = τ_0_ *n* ^–λ^.^70^

### SCLC Measurements

Devices were fabricated as follows: ITO/PEDOT:PSS/active layer/Au for holes and ITO/ZnO/active layer/Al for electrons. The charge carrier mobility was determined by fitting the dark current to the model of a single carrier SCLC according to the equation *J* = (9/8)­με_r_ε_0_ *V* ^2^ exp­(0.89­(*V*/*E*_0_d)^0.5^)/*d*.^3,52^ Here, *J* is the current density, ε_r_ is the relative dielectric constant of the transport medium, ε_0_ is the permittivity of free space, *d* is the film thickness of the active layer, and μ is the charge carrier mobility. *V* = *V*_app_ – *V*_bi_, where *V*_app_ is the applied voltage and *V*_bi_ is the offset voltage. *E*_0_ is the characteristic field, and *d* is the thickness of the active layer.

### Capacitance Spectroscopy Measurements

The chemical capacitance of the device is obtained by subtracting low-frequency *C*_cor_ measured under light with *C*_cor_ measured at −2 V under dark. Measurements are taken at various *V*_b_ from 0 to 0.9 V to obtain *C*_μ_ as a function of *V*_cor_, from which charge carrier density can be obtained using the following equation.

$$n\left(V_{cor}\right)=n_{sat}+\frac{1}{qAL}\int_{Vsat}^{V_{cor}}C_{\mu }dV_{cor}$$

$$n_{sat}=\frac{1}{qAL}C_{sat}\left(V_{0}-V_{sat}\right)$$

*C*_sat_ is the chemical capacitance measured at *V*_sat_ (−2 V) under 1 sun. *V*_0_ is the forward bias at which the photocurrent equals zero.

Effective charge carrier mobilities, carrier extraction lifetimes, and nongeminate recombination lifetimes at various bias conditions can be obtained via the following equations.

$$\mu_{eff}\left(n,V_{cor}\right)=\frac{J\left(V_{cor}\right)\cdot L}{2qn\left(V_{cor}\right)\cdot \left[V_{cor}-V_{oc}\right]}$$

where *L* is the thickness of the active layer and *J*_rec_ is the difference between saturated *J*_ph_ at −2 V and *J*_ph_ at various bias conditions.^71^

## Supplementary Material



## Notes

JV, EQE, AFM, c-AFM, GIWAXS, GISAXS, GISANS, and UPS measurements; TPV and CE analyses; SCLC and capacitance spectroscopy analysis; synthesis procedures and NMR/HRMS spectra of d-BTR-Cl; and supplementary notes and equations (PDF)
