# Supplementary material for: Resolving Ternary Morphology for High-Performance Thickness-Insensitive Organic Solar Cells
Source: ACS Appl Mater Interfaces. 2025 Nov 28;17(49):66924–35. doi: 10.1021/acsami.5c17321 (PMC12874358; doi:10.1021/acsami.5c17321)
Supplement: Supplementary file 1 [file am5c17321_si_001.pdf]

Supporting Information:

Resolving Ternary Morphology for High-Performance Thickness-insensitive Organic Solar Cells

Heng Liu,[‡,1] Yuhao Li,[‡,1,2] Zhaoyang Nie,[‡,3] Yuang Fu,[1] Zhaozhao Bi,[4] Shengtian Zhu,[5] Lu Chen,[6] Guilong Cai,[7] Pok Fung Chan,[1] Luo Huang,[3] Luhang Xu,[1] Chun-Jen Su,[8] U-Ser Jeng,[8] Guangye Zhang,[6] Fei Huang,[5] Wei Ma,[4] Xian-Kai Chen,[9] Yubin Ke,[,2] Man Chung Tang,[*,3] Xinhui Lu[*,1]*

[1]Department of Physics, The Chinese University of Hong Kong, New Territories 999077, Hong Kong, China

[2]Spallation Neutron Source Science Center, Institute of High Energy Physics, Chinese Academy of Sciences, Dongguan 523803, China

[3]Institute of Materials Research, Tsinghua Shenzhen International Graduate School, Tsinghua University, 518055 Shenzhen, China.

[4]State Key Laboratory for Mechanical Behavior of Materials, Xi'an Jiaotong University, Xi'an 710049, China

[5]Institute of Polymer Optoelectronic Materials and Devices, State Key Laboratory of Luminescent Materials and Devices, South China University of Technology, Guangzhou 510640, P. R. China

[6]College of New Materials and New Energies, Shenzhen Technology University, Shenzhen, China

[7]Beijing Key Laboratory of Ionic Liquids Clean Process, CAS Key Laboratory of Green Process and Engineering, State Key Laboratory of Multiphase Complex System, Institute of Process Engineering, Chinese Academy of Sciences, No. 1 Zhongguancun North Second Street, Haidian District, Beijing 100190, China

[8]National Synchrotron Radiation Research Center, Hsinchu Science Park, Hsinchu 30076, Taiwan, Department of Chemical Engineering, National Tsing Hua University, Hsinchu 30013, Taiwan

[9]Institute of Functional Nano & Soft Materials (FUNSOM), Jiangsu Key Laboratory of Advanced Negative Carbon Technologies Soochow University, Suzhou, Jiangsu 215123, P. R. China

‡ These authors contributed equally: Heng Liu, Yuhao Li, Zhaoyang Nie

*Corresponding authors:

Xinhui Lu: xinhui.lu@cuhk.edu.hk

Yubin Ke: keyb@ihep.ac.cn

Man Chung Tang: kobetang2021@sz.tsinghua.edu.cn

Synthesis and characterizations of d-BTR-Cl

Compound 2: To a stirred solution of compound **1** (1.782 g, 6.85 mmol) in THF (45 mL) was added *n*-BuLi (3.3 mL, 2.5 M in Hexane, 8.25 mmol) at −78 °C. The mixture was allowed to stir at that temperature for 1 h before $B(O^iPr)_3$ (1.691 g, 8.99 mmol) was added. The resultant mixture was allowed to warm to 0 °C and stir at that temperature for 1 h. Pinacol (1.702 g, 14.4 mmol) was then added and stirred for 6 h at room temperature before saturated aq. NH_4Cl (100 mL) was added. The organic layer was separated, and the aqueous layer was extracted with dichloromethane (3 × 50 mL). The combined organic phases were washed with brine (50 mL), dried over anhydrous Na_2SO_4, filtered, and concentrated under vacuum. The residue was subjected to flash column chromatography for purification using dichloromethane /petroleum ether (1:8) to give compound **2** (1.370 g, 65%) as a yellow liquid. ^{1}H NMR (400 MHz, $CDCl_3$): δ = 7.49 (d, *J* = 4.6 Hz, 1 H), 7.01 (d, *J* = 4.6 Hz, 1 H), 1.33 (s, 12 H) ppm; ^{13}C NMR (101 MHz, $CDCl_3$): δ = 154.84, 131.42, 130.41, 83.64, 24.91 ppm; HRMS (*m/z*): $[M + H]^+$ calcd for $C_{16}H_{15}D_{13}O_2BS^+$ 308.2714, found 308.2712.

Compound 3: Compound 2 (2.7 g, 4.4 mmol), Compound S1 (681 mg, 2.22 mmol) and $Pd(PPh_3)_4$ (224mg, 0.194 mmol), Cs_2CO_3 (1.836 g, 5.63 mmol) were added dry toluene (30 ml) and stirred at 120 °C for 12 h. After cooling down to room temperature, the resulting mixture was filtered by celite and washed with ethyl acetate (80 ml). The filtrate was concentrated under vacuum. The residue was subjected to flash column chromatography for purification using ethyl acetate /petroleum ether (1:12) to give compound **3** (565 mg, 87%) as a yellow liquid. ^{1}H NMR (400 MHz, $CDCl_3$): δ = 7.90 (d, *J* = 4.0 Hz, 1 H), 7.21 (d, *J* = 5.2 Hz, 1 H), 7.10 (d, *J* = 4.0 Hz, 1 H), 6.95 (d, *J* = 5.2 Hz, 1 H), 3.80 (s, 3 H), 3.38 (s, 3

H) ppm; ^{13}C NMR (101 MHz, $CDCl_3$): δ = 162.35, 143.39, 140.86, 134.93, 132.02, 130.38, 129.96, 125.42, 124.83, 61.72, 33.19 ppm; HRMS (*m/z*): $[M + H]^+$ calcd for $C_{17}H_{11}D_{13}NO_2S_2^+$ 301.2059, found 351.2050.

Compound 4: To a stirred solution of compound **3** (575 mg, 1.64 mmol) and acetic acid (2.0 ml) in $CHCl_3$ (10 mL) was added NBS (299 mg1.68 mmol) at room temperature. The mixture was allowed to stir at that temperature for 3 h before saturated aq. $NaHCO_3$ (10 mL) was added. The organic layer was separated, and the aqueous layer was extracted with dichloromethane (3 × 20 mL). The combined organic phases were washed with brine (10 mL), dried over anhydrous Na_2SO_4, filtered, and concentrated under vacuum. The residue was subjected to flash column chromatography for purification using ethyl acetate /petroleum ether (1:12) to give compound **4** (570 mg, 81%) as a yellow liquid. ^1H NMR (400 MHz, $CDCl_3$): δ = 7.88 (d, J = 3.9 Hz, 1 H), 7.04 (d, J = 4.0 Hz, 1 H), 6.91 (s, 1 H), 3.79 (s, 5 H), 3.38 (s, 5 H) ppm; ^{13}C NMR (101 MHz, $CDCl_3$): δ = 162.12, 141.87, 141.43, 134.92, 133.01, 132.49, 131.44, 125.75, 111.71, 61.75, 33.18 ppm; HRMS (*m/z*): $[M + H]^+$ calcd for $C_{17}H_{10}D_{13}BrNO_2S_2^+$ 429.1164, found 429.1159.

Compound 5: To a mixture of compound **2** (181 mg, 0.589 mmol), compound **4** (200 mg, 0.466 mmol) , K_2CO_3 (197 mg, 1.43 mmol) and $Pd(PPh_3)_4$ (56 mg, 0.485 mmol) was added dimethoxymethane (5.0 ml). The resulting suspension was sealed and heated to 120 °C for 1 h by microwave heating. After cooling down to room temperature, the suspension was filtered by celite and washed with ethyl acetate (50 ml). The filtrate was concentrated under vacuum. The residue was then subjected to flash column chromatography for purification using ethyl acetate /petroleum ether (1:12) to give compound **5** (161 mg, 61%) as a yellow liquid. ^1H NMR (400 MHz, $CDCl_3$): δ = 7.95–7.89 (m, 1 H), 7.20–7.12 (m, 1 H), 7.15–

7.11 (m, 1 H), 6.98–6.94 (m, 1 H), 6.96–6.90 (m, 1 H), 3.81 (s, 3 H), 3.39 (s, 3 H) ppm; ^{13}C NMR (101 MHz, $CDCl_3$): δ = 162.30, 143.06, 141.09, 140.01, 135.49, 134.99, 131.88, 130.26, 129.71, 128.94, 125.15, 124.00, 61.72, 33.19 ppm; HRMS (m/z): $[M + H]^+$ calcd for $C_{27}H_{12}D_{26}NO_2S_3^+$ 530.3691, found 530.3680.

Compound 6: To a stirred solution of compound **5** (436 mg, 0.823 mmol) and acetic acid (1.0 ml) in $CHCl_3$ (5 mL) was added NBS (151 mg, 0.848 mmol) at room temperature. The mixture was allowed to stir at that temperature for 3 h before saturated aq. $NaHCO_3$ (20 mL) was added. The organic layer was separated, and the aqueous layer was extracted with dichloromethane (3 × 20 mL). The combined organic phases were washed with brine (20 mL), dried over anhydrous Na_2SO_4, filtered, and concentrated under vacuum. The residue was subjected to flash column chromatography for purification using ethyl acetate /petroleum ether (1:12) to give compound **6** (411 mg, 82%) as a yellow liquid. ^{1}H NMR (400 MHz, $CDCl_3$): δ = 7.91 (d, 1 H), 7.12 (d, J = 4.0 Hz, 1 H), 6.89 (d, J = 4.0 Hz, 2 H), 3.81 (s, 3 H), 3.39 (s, 3 H) ppm; ^{13}C NMR (101 MHz, $CDCl_3$): δ = 162.24, 142.72, 141.13, 140.58, 135.00, 134.05, 132.87, 132.14, 131.80, 130.27, 129.29, 125.35, 110.74, 61.75, 33.20 ppm; HRMS (*m/z*): $[M + H]^+$ calcd for $C_{27}H_{11}D_{26}BrNO_2S_3^+$ 608.2796, found 608.2783.

Compound 7: To a stirred solution of compound **6** (283 mg, 0.465 mmol) in THF(4.7 ml) was added DIBAL-H (0.38 mL, 1.5 M in toluene, 0.570 mmol) at −78 °C. The mixture was allowed to stir at that temperature for 3 h before saturated aq. NH_4Cl (20 mL) was added. The resultant mixture was extracted with ethyl acetate (3 × 10 mL). The combined organic phases were washed with brine (10 mL), dried over anhydrous Na_2SO_4, filtered, and concentrated under vacuum. The residue was subjected to flash column chromatography for purification using dichloromethane /petroleum ether (1:2) as eluent to give compound

7 (215 mg, 84%) as a yellow liquid. ^{1}H NMR (400 MHz, $CDCl_3$): δ = 9.88 (s, 1 H), 7.70 (d, *J* = 4.0 Hz, 1 H), 7.22 (d, *J* = 4.0 Hz, 1 H), 6.90 (d, *J* = 5.5 Hz, 2 H) ppm; ^{13}C NMR (101 MHz, $CDCl_3$): δ = 182.72, 146.07, 142.56, 142.46, 141.02, 136.96, 135.26, 133.00, 131.46, 129.83, 129.54, 126.13, 111.24 ppm; HRMS (*m/z*): $[M + H]^+$ calcd for $C_{25}H_6D_{26}BrOS_3^+$ 549.24361, found 549.2415.

Compound 8: A mixture of compound **7** (127 mg, 0.231 mmol), compound **S2** (93 mg, 0.0924 mmol) and $Pd(PPh_3)_4$ (55 mg, 0.476 mmol) was dissolved in dry toluene (5.0 ml). After cooling down to room temperature, the solution was concentrated under vacuum. The residue was subjected to flash column chromatography for purification using toluene as eluent to give compound **8** (121 mg, 81%) as a red foam.^{1}H NMR (600 MHz, $CDCl_3$): δ = 9.88 (s, 2 H), 7.71 (d, *J* = 3.9 Hz, 2 H), 7.55 (s, 2 H), 7.25 (s, 2 H), 7.23 (d, *J* = 3.9 Hz, 2 H), 7.12 (s, 2 H), 7.02 (s, 2 H) ppm; ^{13}C NMR (101 MHz, $CDCl_3$): δ = 182.70, 146.18, 142.69, 142.34, 141.31, 138.83, 138.70, 138.17, 137.42, 137.00, 136.01, 135.61, 135.22, 130.92, 129.75, 129.25, 128.81, 128.15, 125.99, 122.99, 122.56, 118.63 ppm; HRMS (*m/z*): $[M + H]^+$ calcd for $C_{84}H_{15}D_{86}Cl_2OS_{10}^+$ 1617.9778, found 1617.9689.

***d*-BTR-Cl:** To a stirred solution of compound **8** (70 mg, 0.432 mmol) and compound **S3** (98 mg. 0.451 mmol) in $CHCl_3$ (8 mL) was added DBU (15 mg, 0.0985 mmol) at room temperature. The mixture was allowed to stir at that temperature for 3 h. The resulting solution was then concentrated under vacuum. The residue was subjected to flash column chromatography for purification using toluene as eluent to give ***d*-BTR-Cl** (215 mg, 84%) as a dark red solid.^{1}H NMR (600 MHz, $CDCl_3$): δ = 7.79 (s, 2 H), 7.48 (s, 2 H), 7.31 (d, *J* = 4.1 Hz, 2 H), 7.24 (s, 2 H), 7.15 (d, *J* = 3.9 Hz, 2 H), 7.06 (s, 2 H), 6.96 (s, 2 H), 4.09–4.04 (m, 4 H), 1.69 (m, 4 H), 1.38–1.28 (m, 12 H), 0.91–0.85 (m, 6 H) ppm; ^{13}C NMR (101 MHz, $CDCl_3$): δ = 192.09, 167.44, 144.23, 141.85, 140.92, 138.58, 138.46, 137.91, 137.19, 137.00, 135.50, 134.89,

134.57, 130.91, 129.77, 128.99, 128.60, 128.00, 126.38, 124.87, 122.82, 122.27, 120.18, 118.38, 44.84, 31.32, 29.69, 26.44, 22.50, 14.00 ppm; HRMS (*m/z*): $[M + H]^+$ calcd for $C_{102}H_{41}D_{86}Cl_2N_2O_2S_{14}^+$ 2016.0757, found 2016.0681.

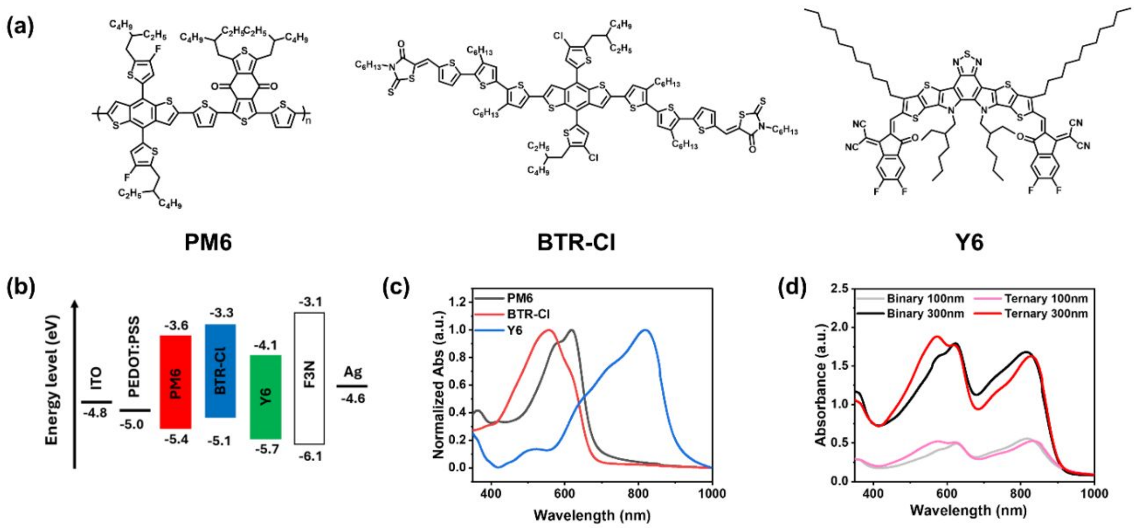

Figure S1. (a) Chemical structure of PM6, BTR-Cl and Y6; (b) Energy level alignment of PM6, BTR-Cl and Y6; (c) Normalized ultraviolet-visible (UV-vis) spectra of neat films; (d) UV-vis spectra of blend films

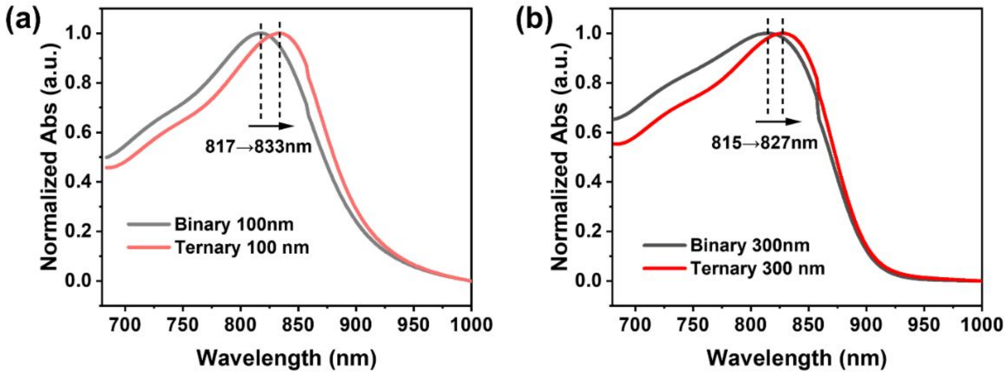

Figure S2. The localized zoomed-in UV–vis absorption spectra of binary and ternary blend films with different thicknesses: (a) 100 nm and (b) 300 nm.

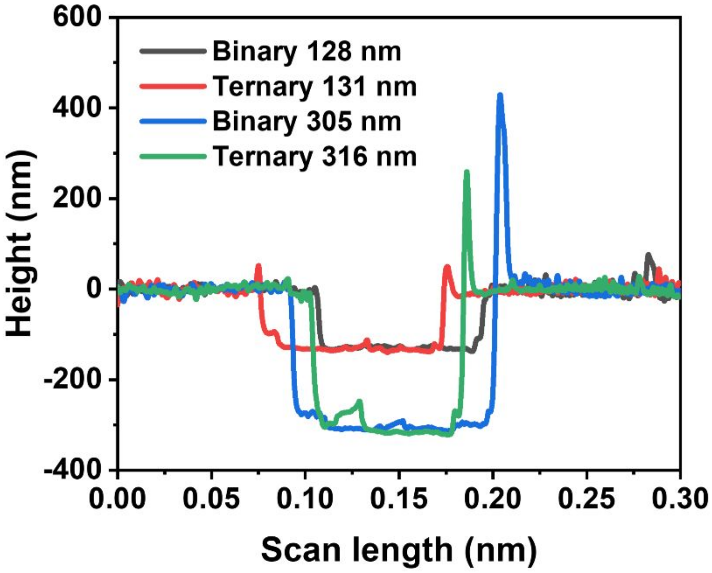

Figure S3. Film thickness measurements of binary and ternary blend films using a surface profilometer.

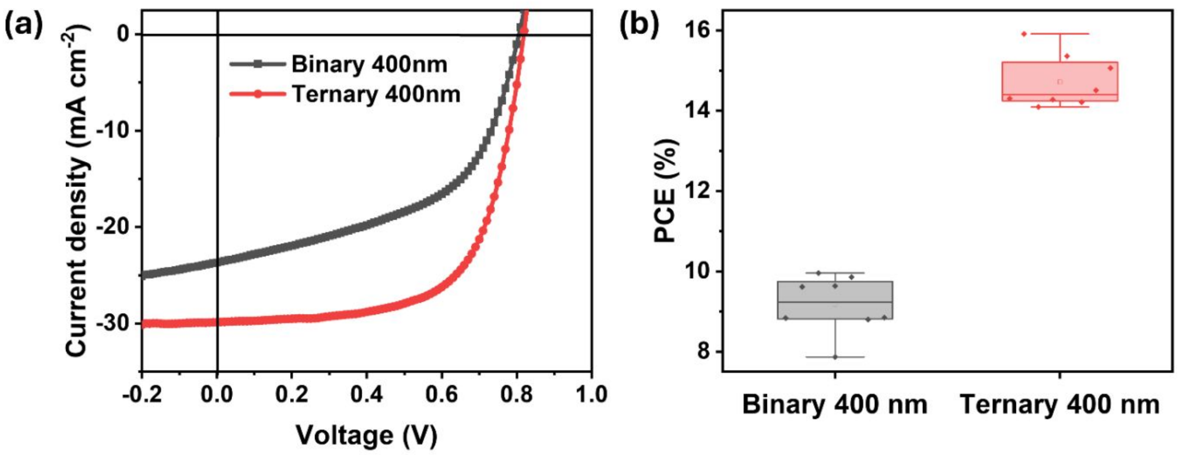

Figure S4. (a) the best J-V curves of binary and ternary 400 nm devices. (b) Statistical histograms of PCE of binary and ternary 400 nm devices.

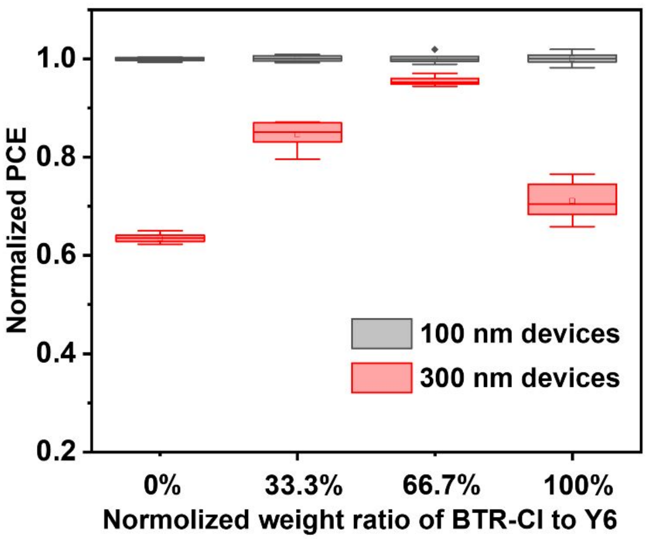

Figure S5. The PCE retention rate versus the weight ratio of BTR-Cl normalized to that of Y6 when the film thickness increases from 100 nm to 300 nm.

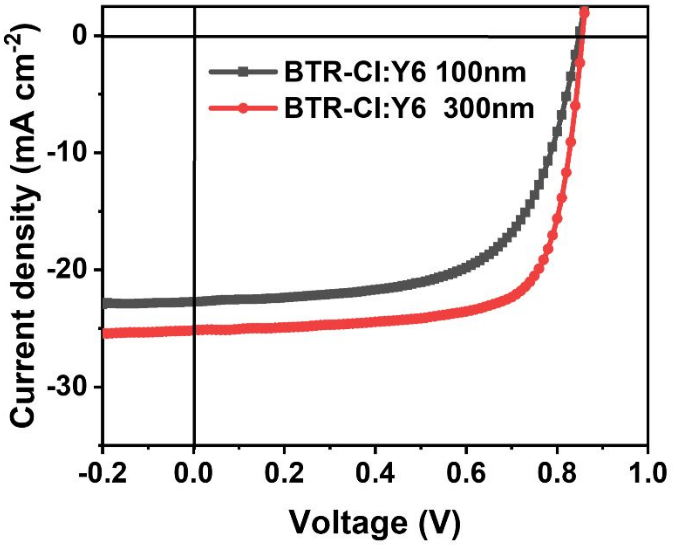

Figure S6. J–V characteristics of BTR-Cl:Y6-based binary devices with active layer thicknesses of 100 nm (black) and 300 nm (red) under AM 1.5G illumination (100 mW cm^{-2}).

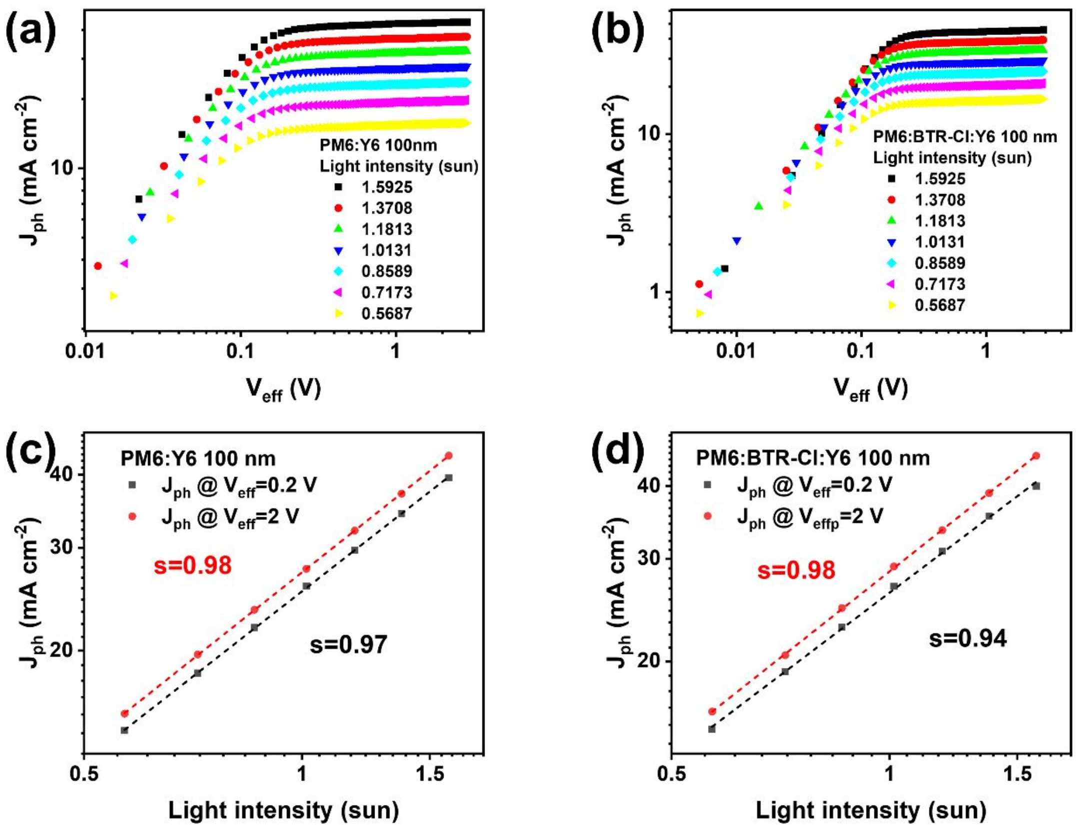

Figure S7. (a, b) Incident light power dependence of the photocurrent versus the effective voltage of thin-film devices; (c, d) Incident light power dependence of the photocurrent extracted at Veff=0.2 V and 2 V of thin-film devices.

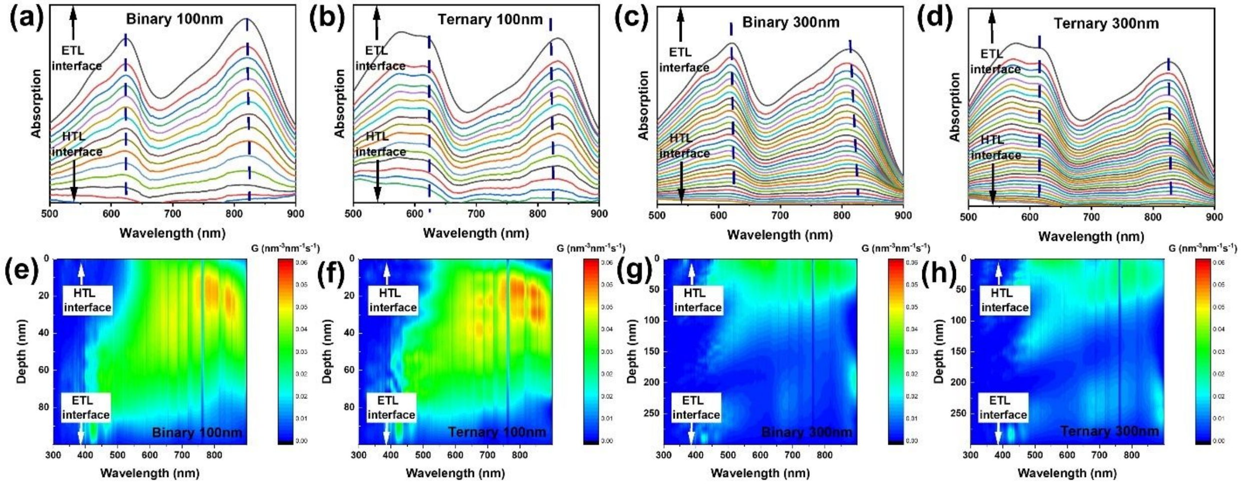

Figure S8. (a-d) Film-depth-dependent absorption spectroscopy and (e-h) Calculated exciton generation contours of binary and ternary blend films.

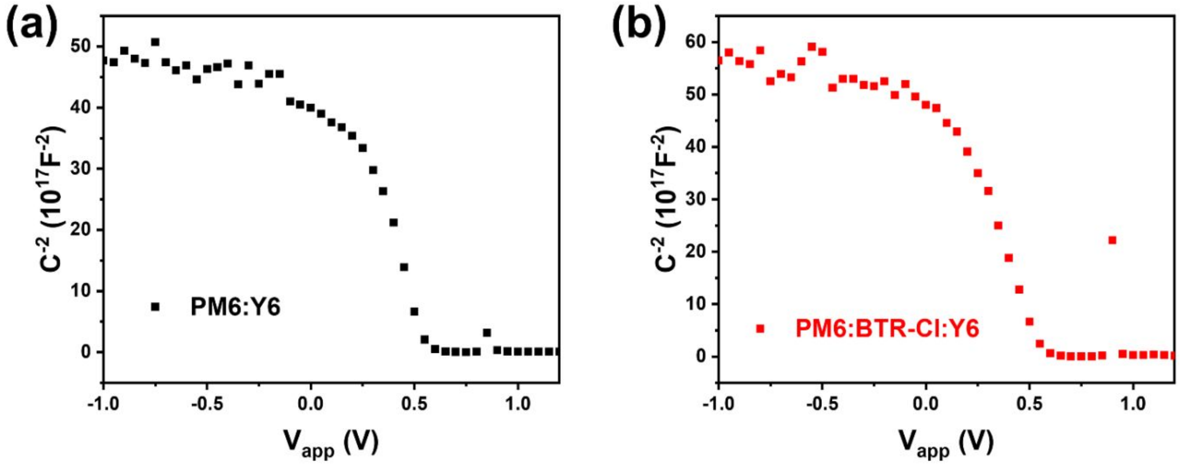

Figure S9. Mott–Schottky analyses for binary (a) and ternary (b) thick films.

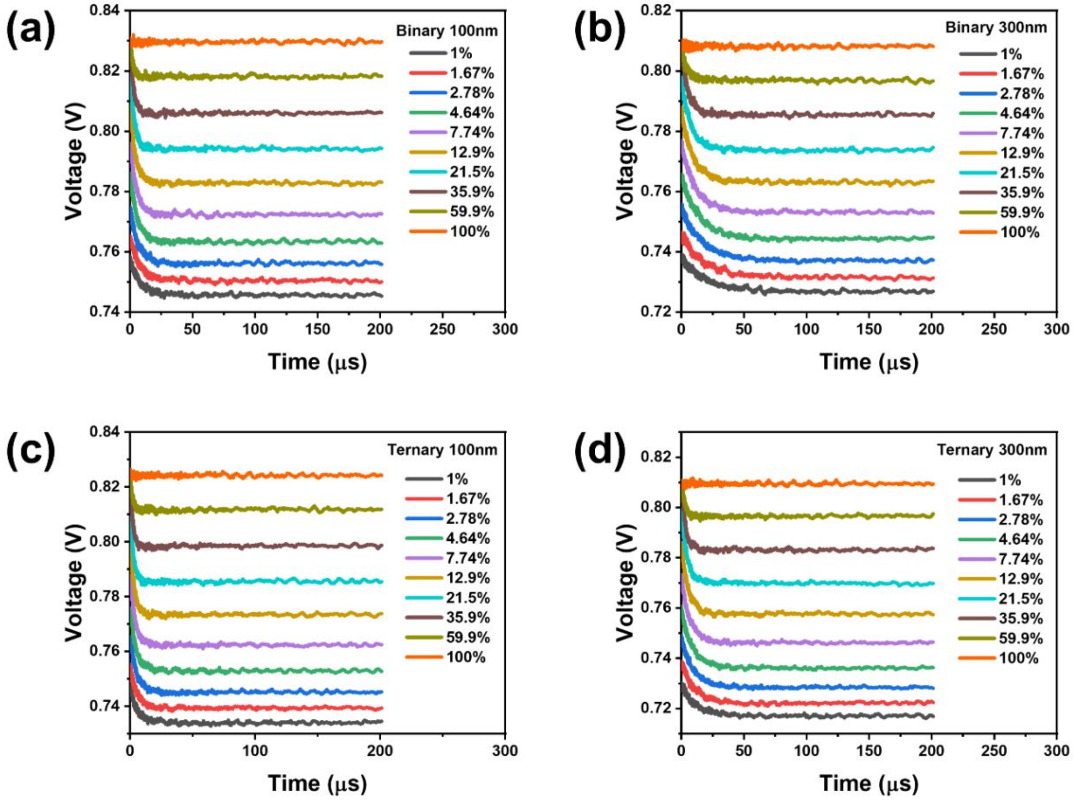

Figure S10. TPV decay dynamics under different illumination intensities of blend films. (a) Binary 100 nm, (b) Binary 300 nm, (c) Ternary 100 nm and (d) Ternary 300 nm.

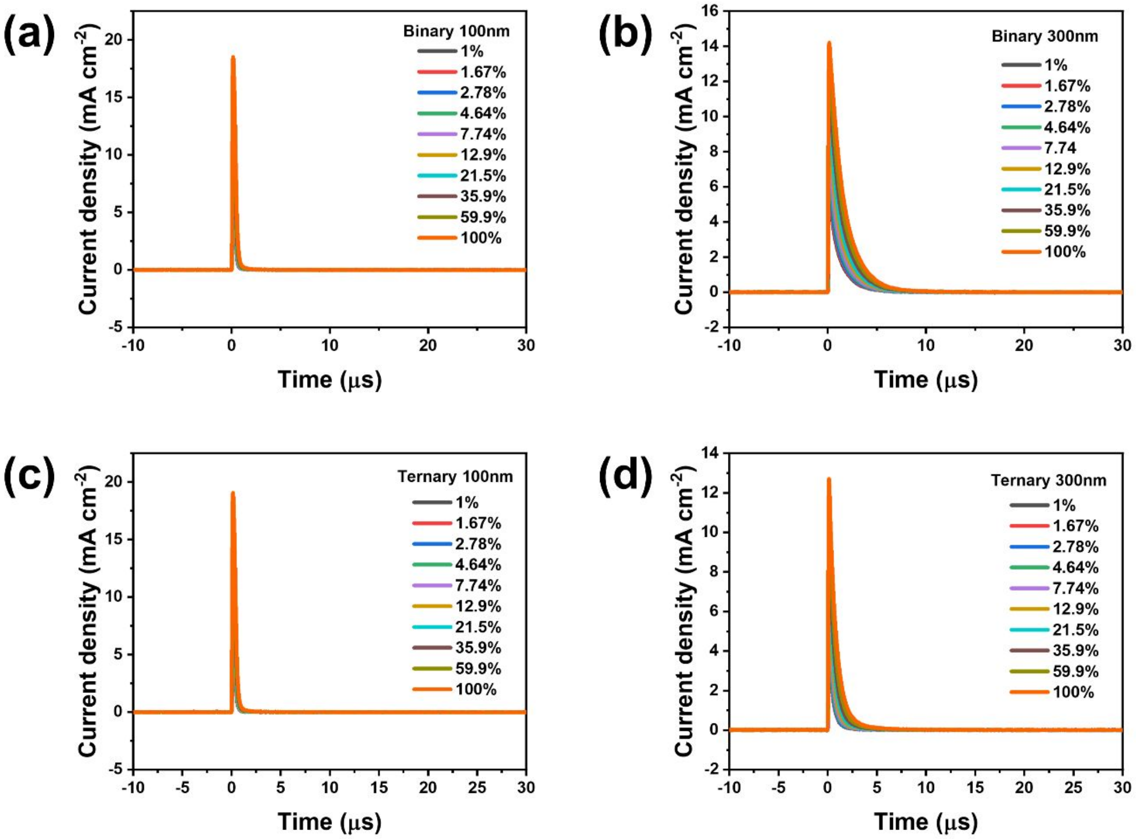

Figure S11. Charge extraction traces under different illumination intensities of blend films. (a) Binary 100 nm, (b) Binary 300 nm, (c) Ternary 100 nm and (d) Ternary 300 nm.

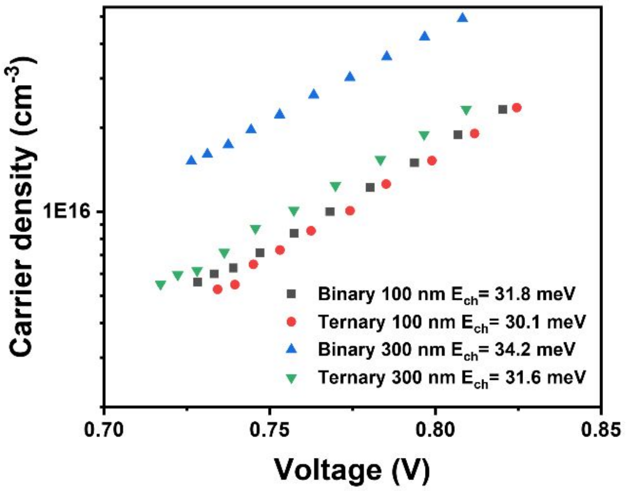

Figure S12. The charge carrier density versus photoinduced open-circuit voltage in the different systems, the slope γ indicates the density of tail states distribution against the energy.

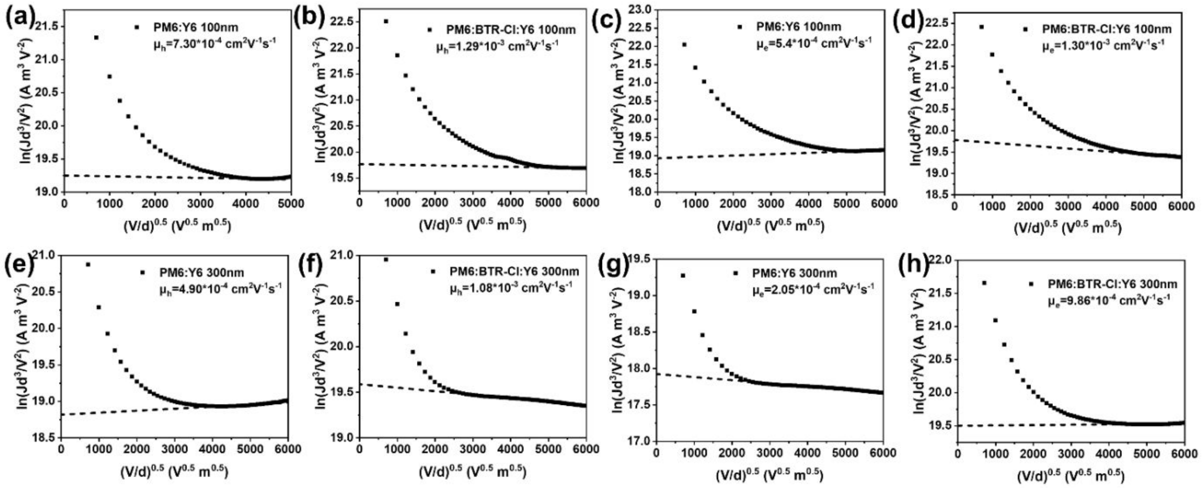

Figure S13. SCLC (space-charge-limited current) fitting curves used to extract charge carrier mobilities in binary and ternary blend films with different thicknesses. (a) Hole-only device for PM6:Y6 (100 nm), (b) Hole-only device for PM6:BTR-Cl:Y6 (100 nm), (c) Electron-only device for PM6:Y6 (100 nm), (d) Electron-only device for PM6:BTR-Cl:Y6 (100 nm), (e) Hole-only device for PM6:Y6 (300 nm), (f) Hole-only device for PM6:BTR-Cl:Y6 (300 nm), (g) Electron-only device for PM6:Y6 (300 nm), (h) Electron-only device for PM6:BTR-Cl:Y6 (300 nm).

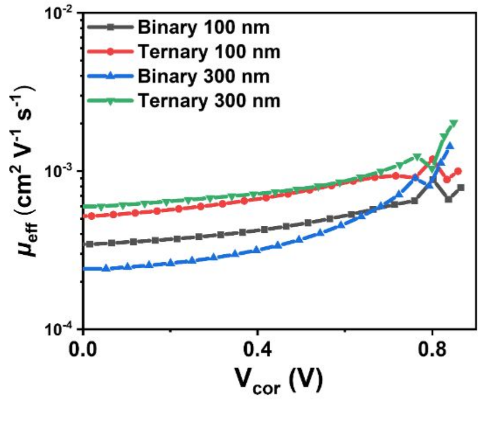

Figure S14. The effective mobilities (μ_{eff}) derived as a function of corrected voltage (V_{cor}) from capacitance spectroscopy

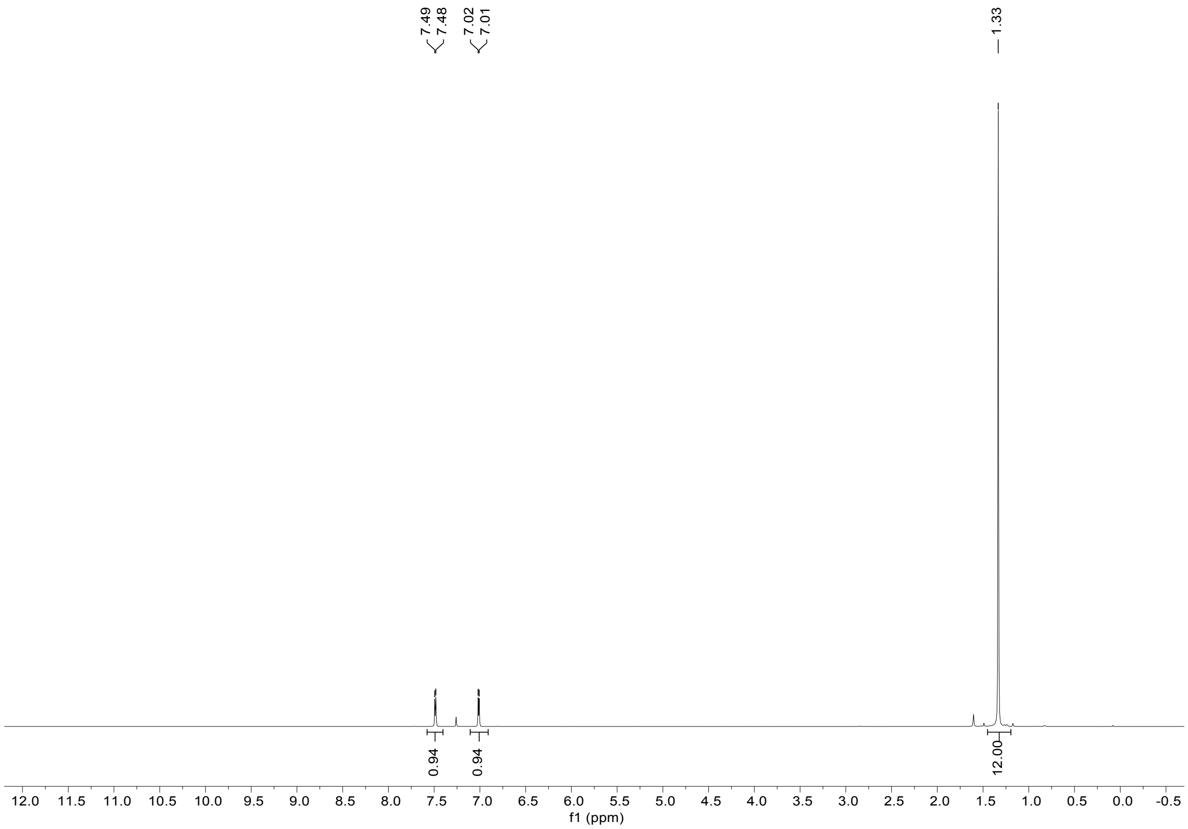

Figure S15. ^{1}H NMR spectrum of compound **2**.

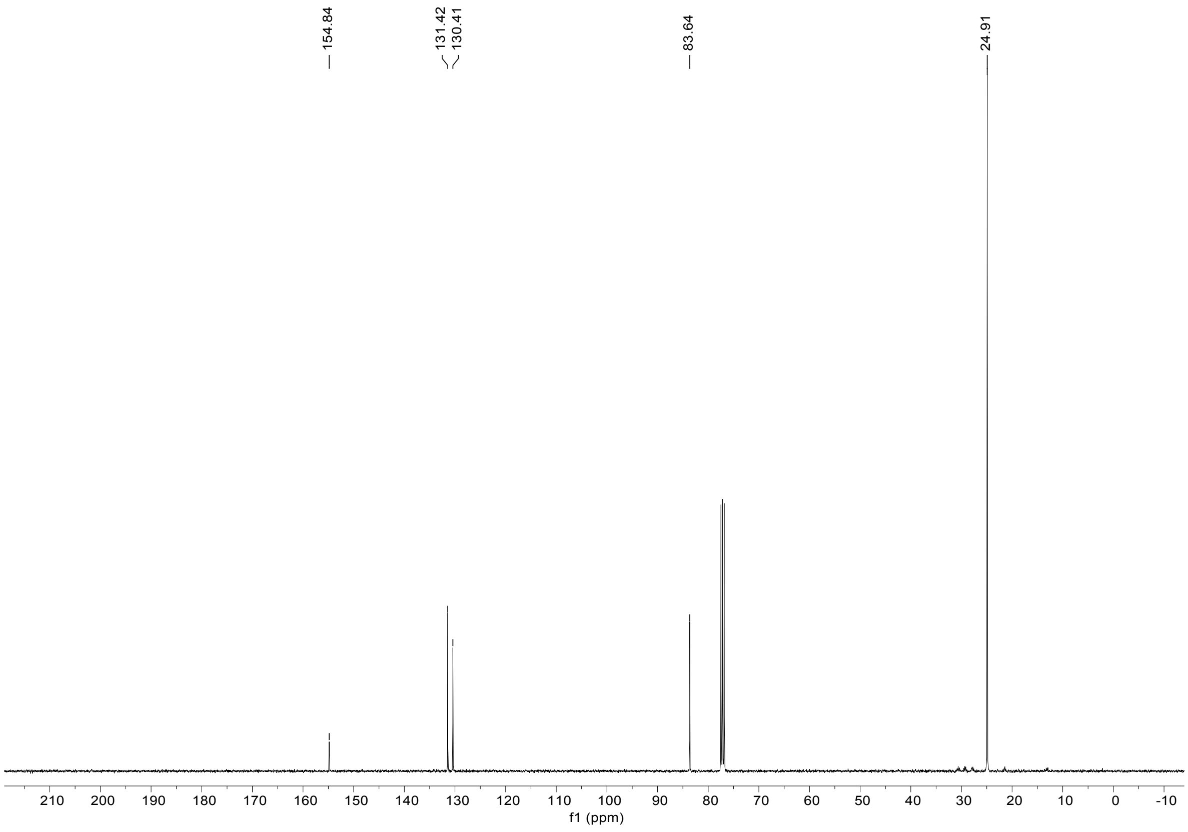

Figure S16. ^{13}C NMR spectrum of compound **2**.

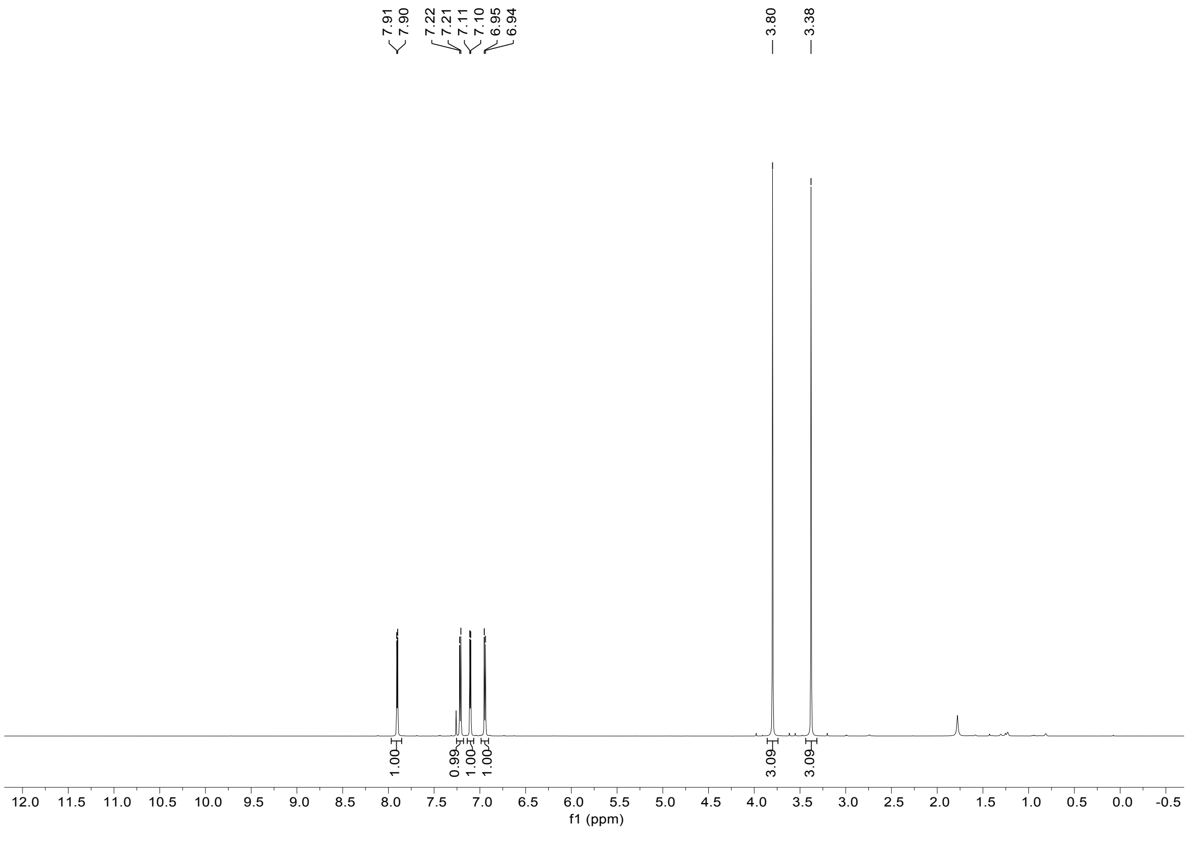

Figure S17. ^{1}H NMR spectrum of compound **3**.

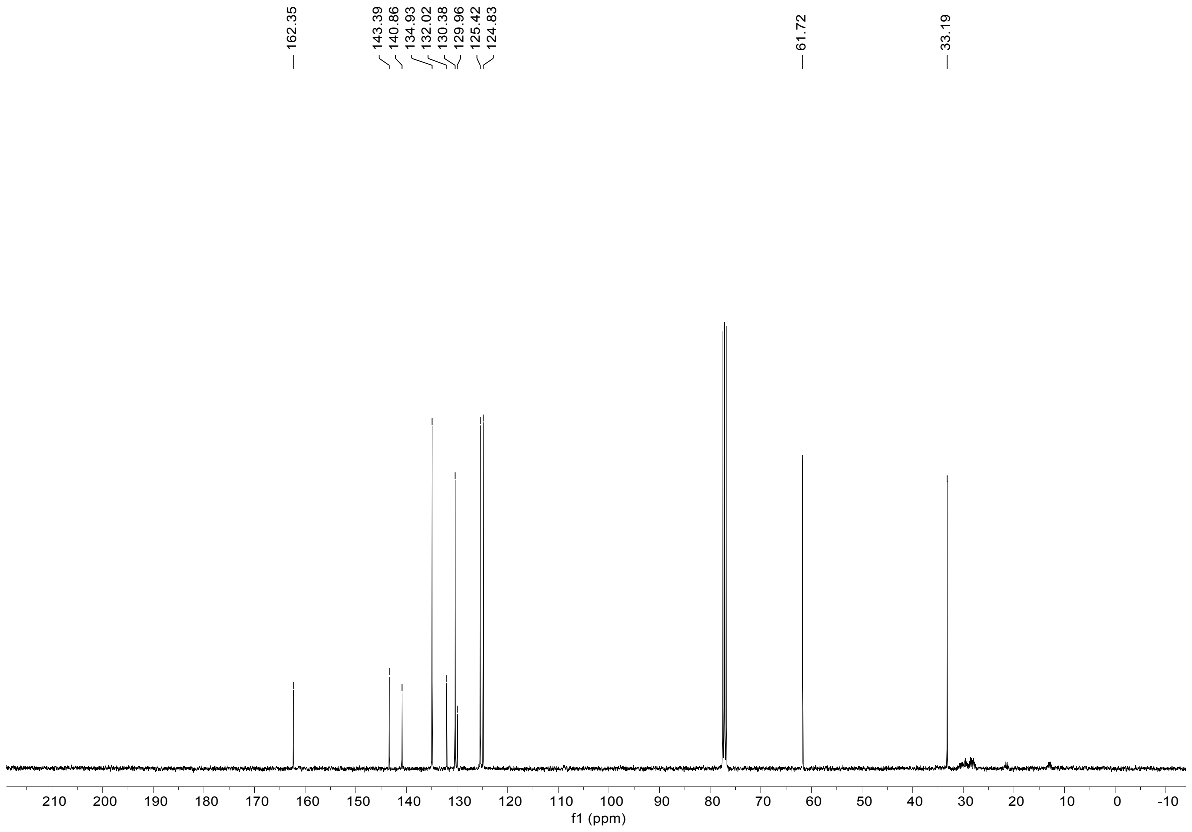

Figure S18. ^{13}C NMR spectrum of compound **3**.

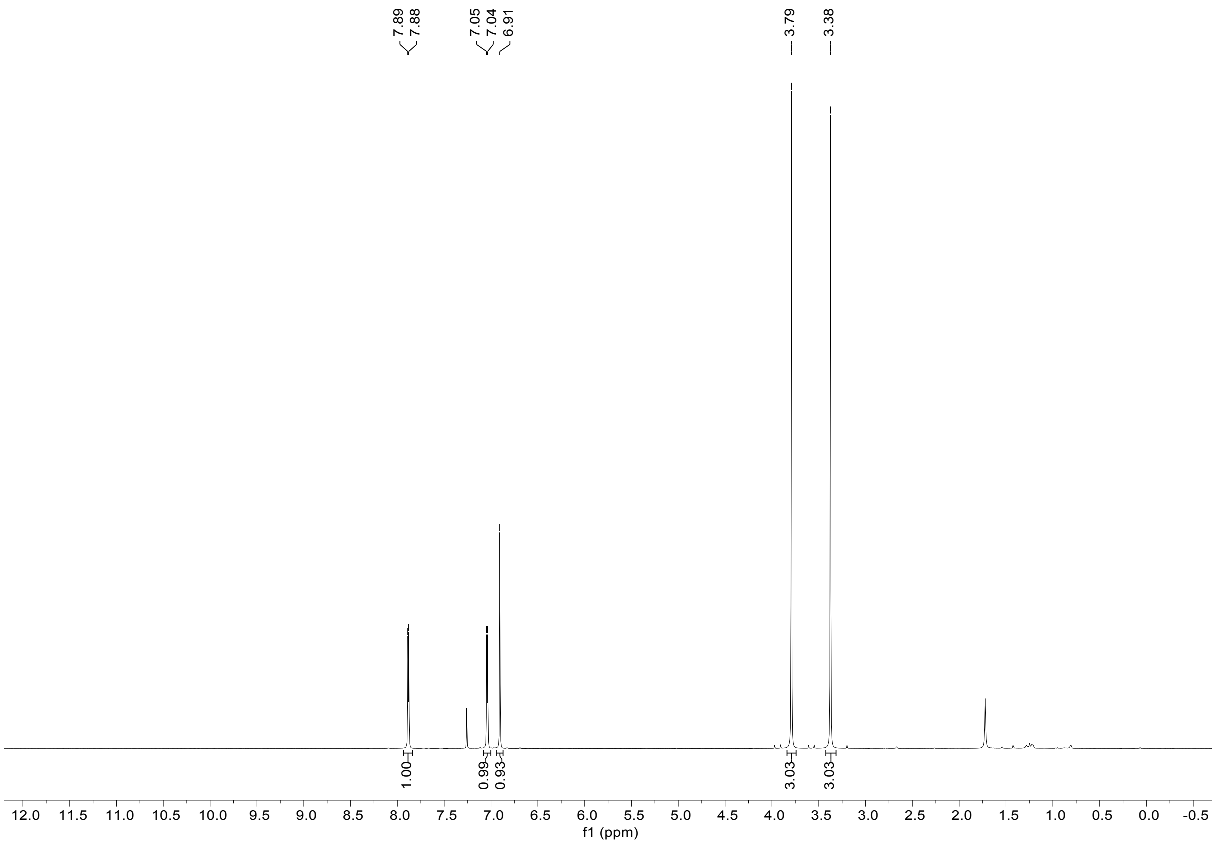

Figure S19. ^{1}H NMR spectrum of compound **4**.

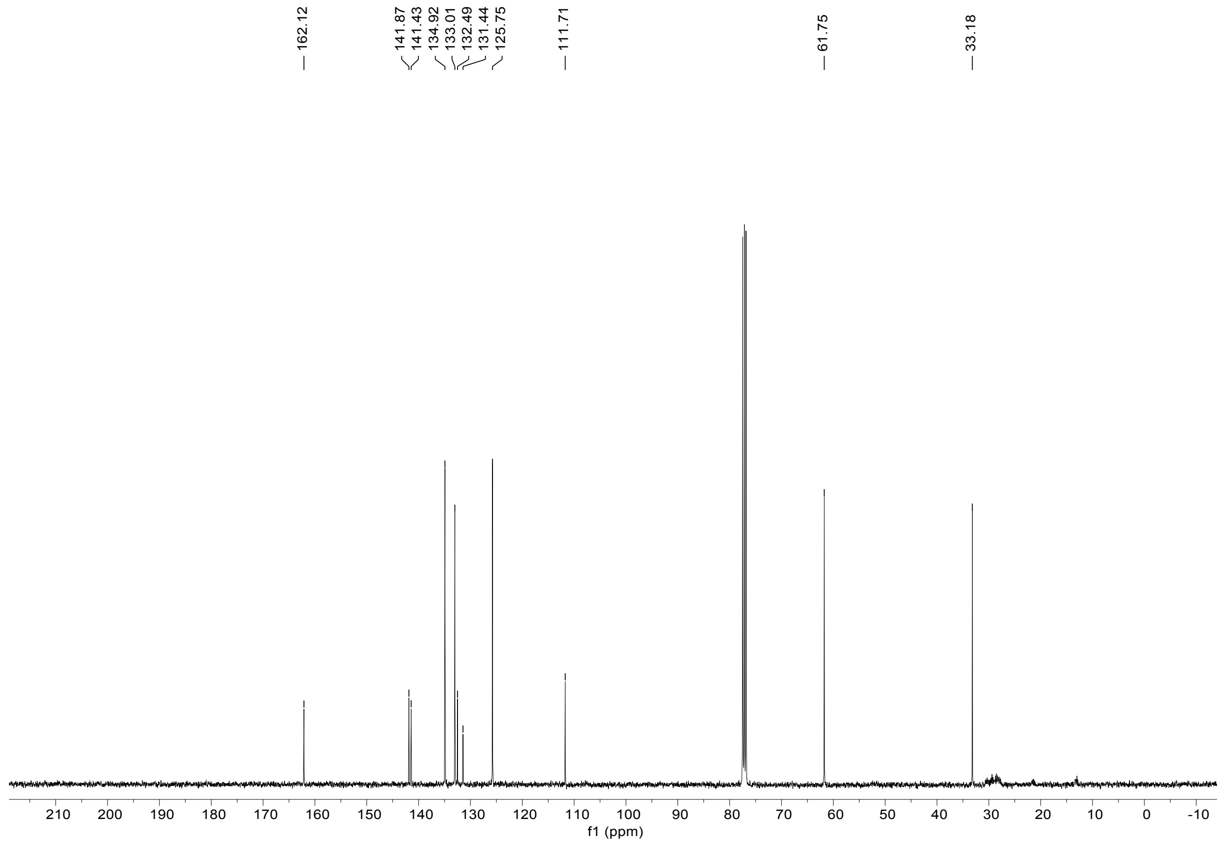

Figure S20. ^{13}C NMR spectrum of compound **4**.

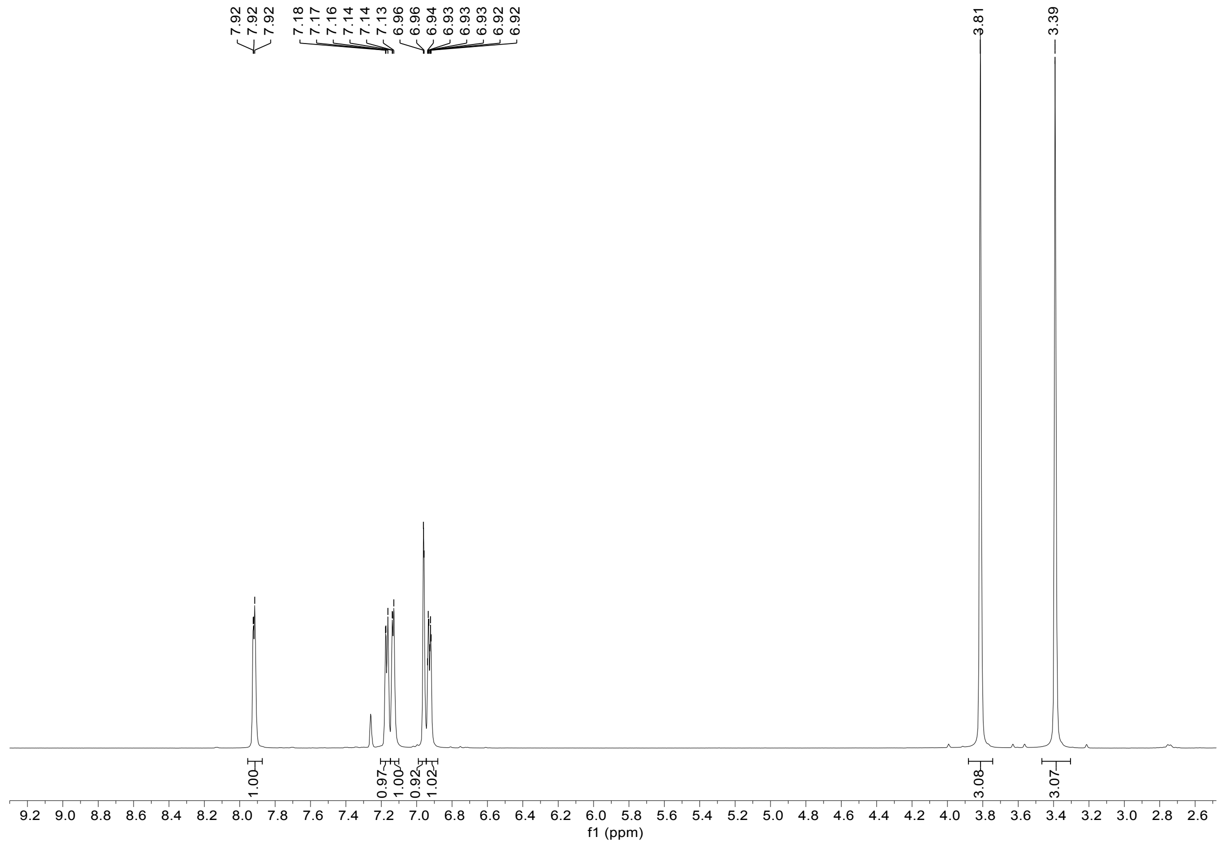

Figure S21. ^{1}H NMR spectrum of compound **5**.

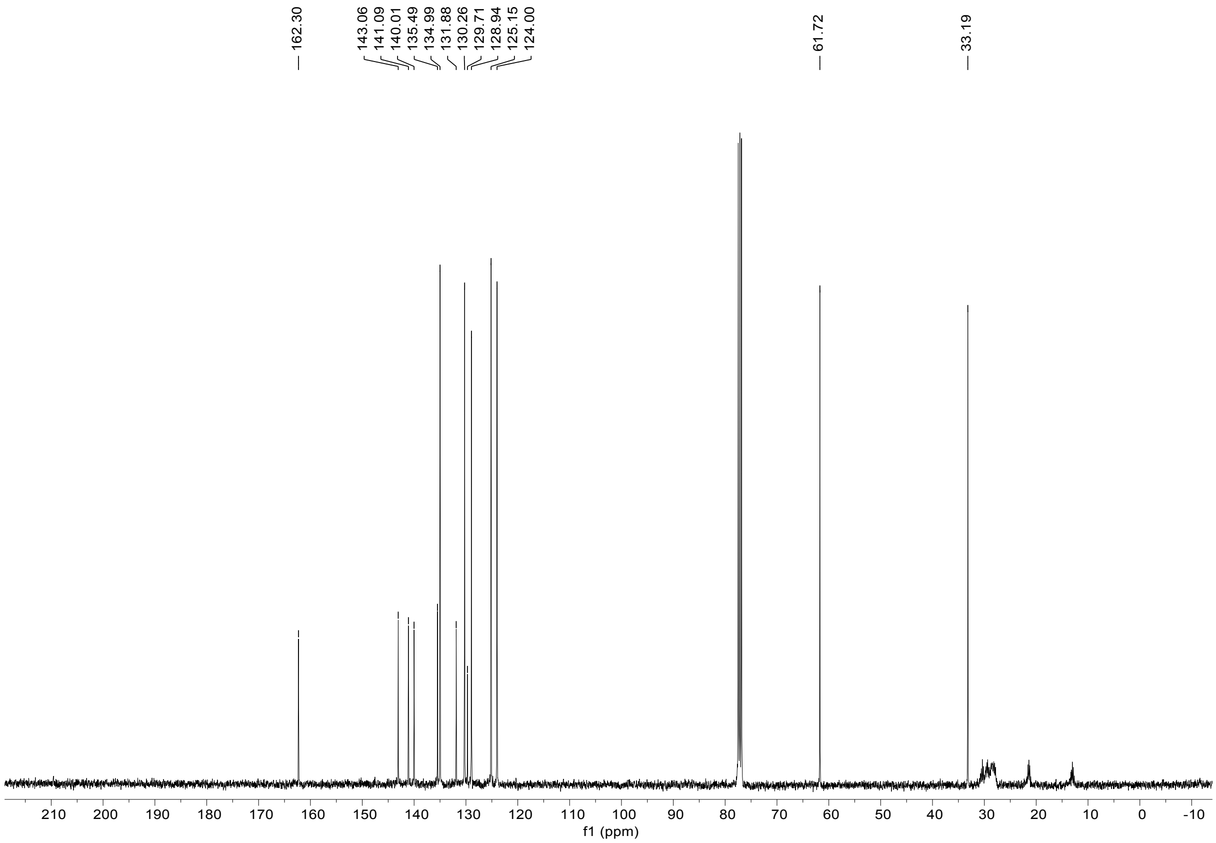

Figure S22. ^{13}C NMR spectrum of compound **5**.

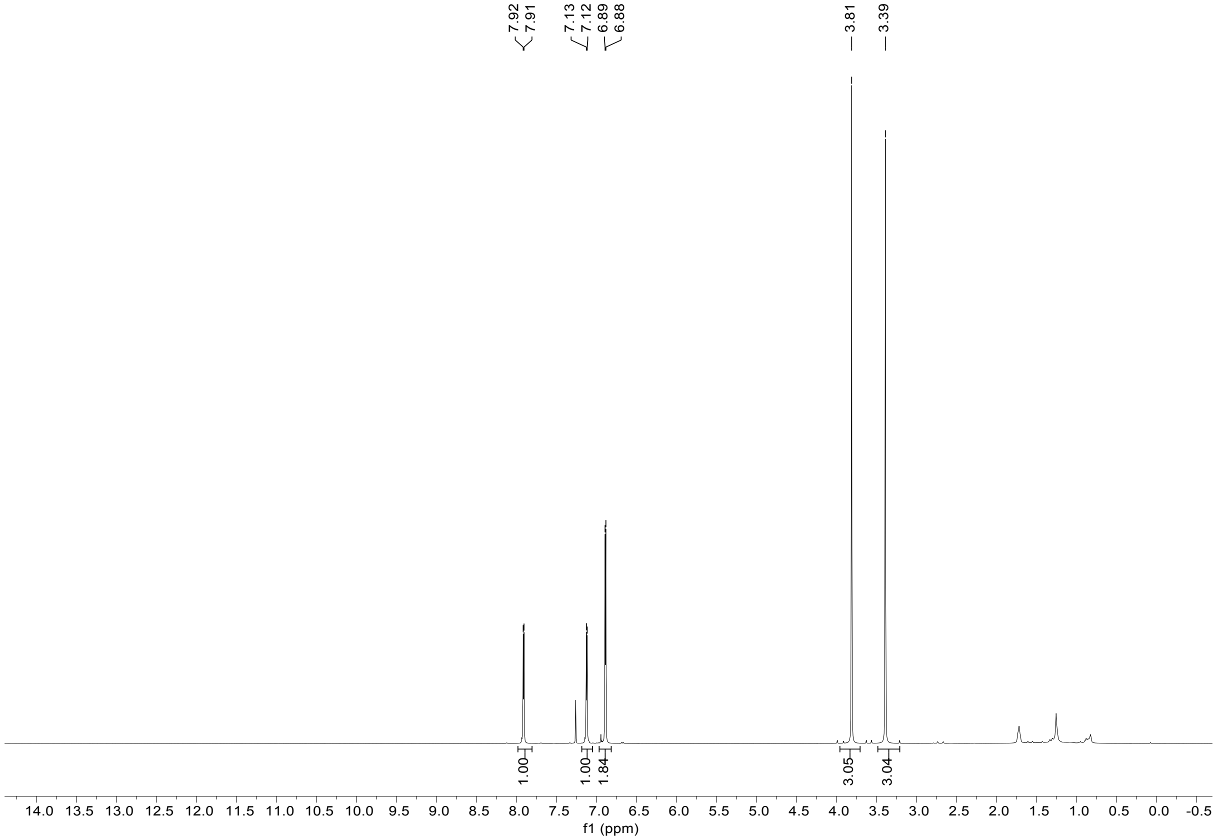

Figure S23. ^{1}H NMR spectrum of compound **6**.

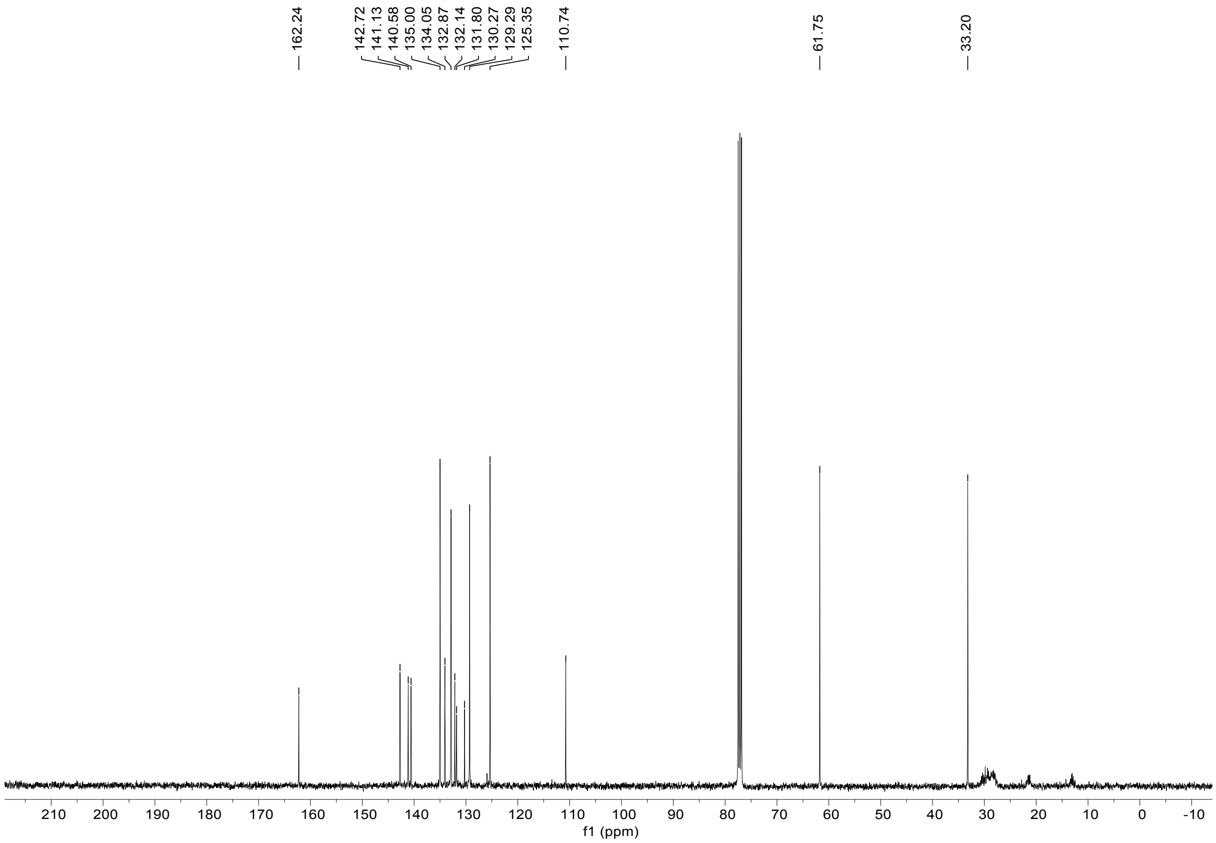

Figure S24. ^{13}C NMR spectrum of compound **6**.

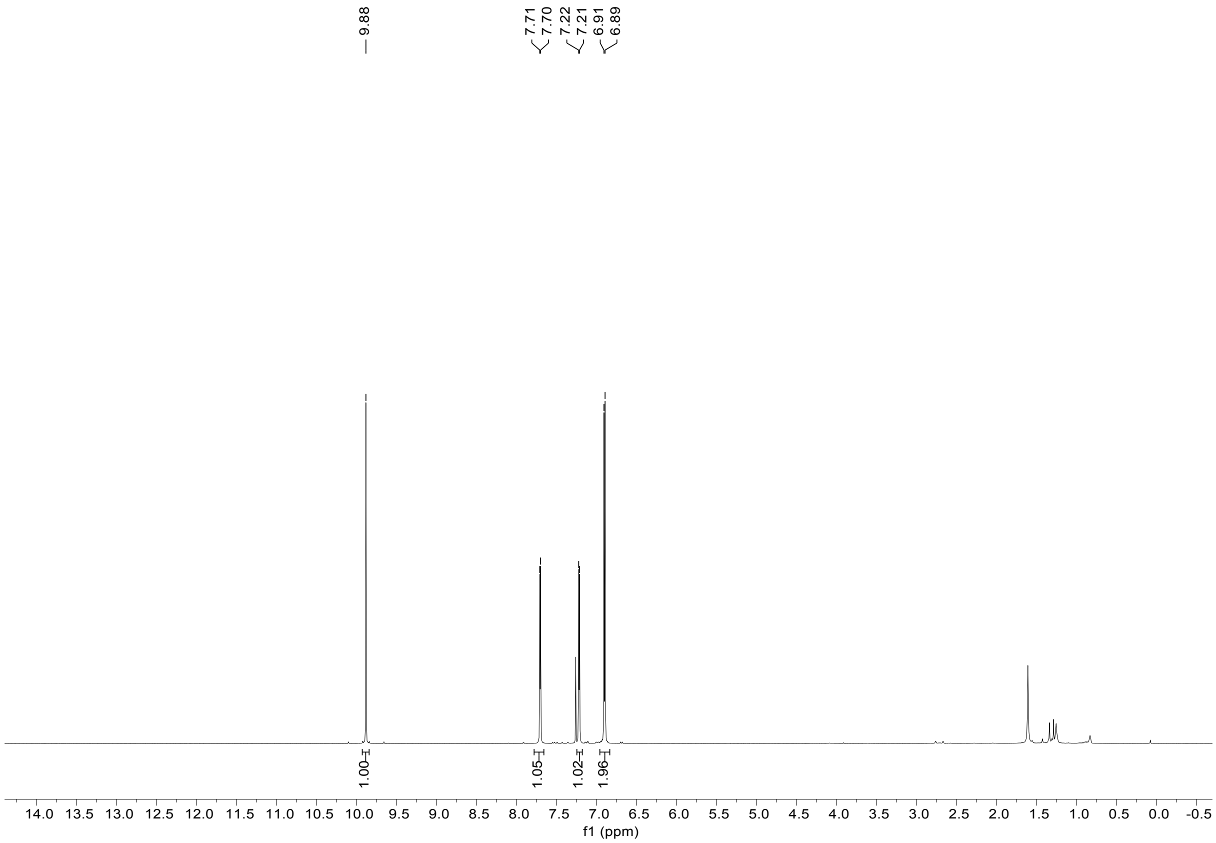

Figure S25. ^{1}H NMR spectrum of compound **7**.

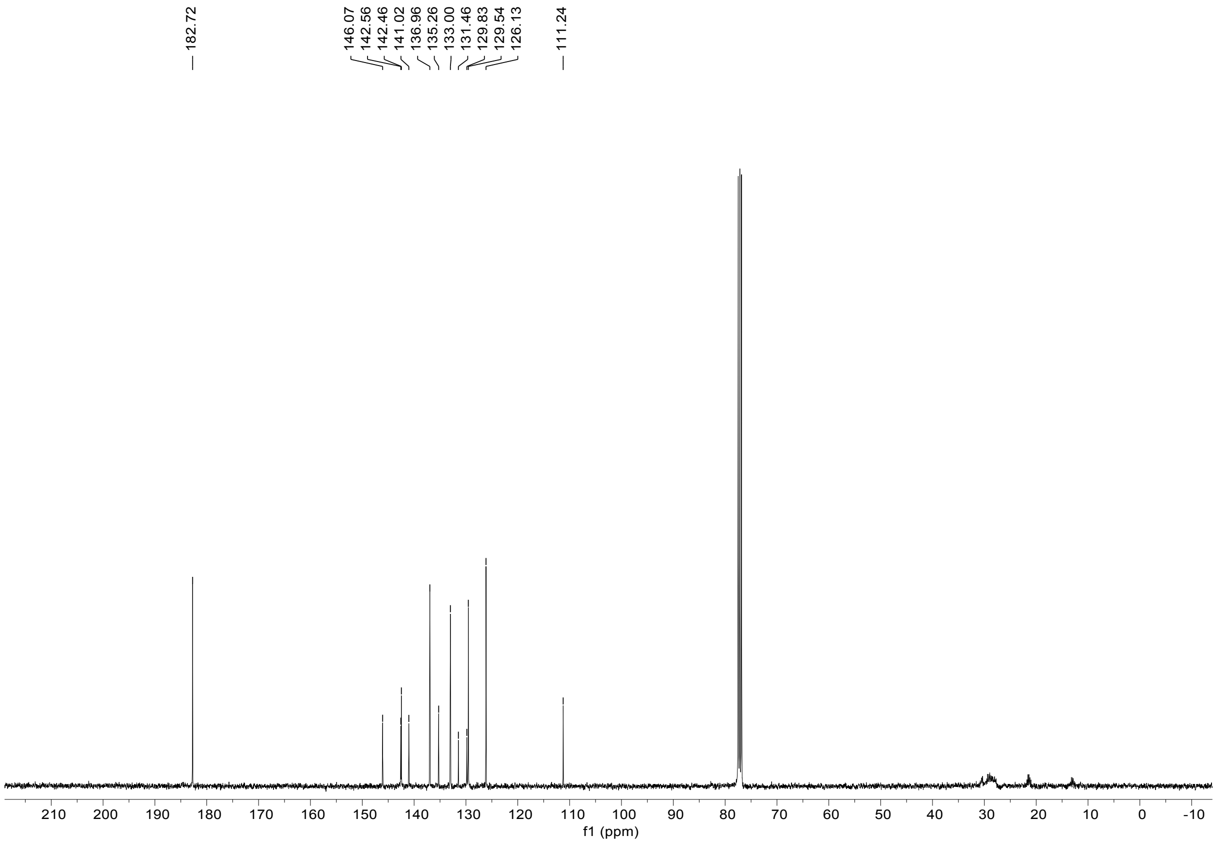

Figure S26. ^{13}C NMR spectrum of compound **7**.

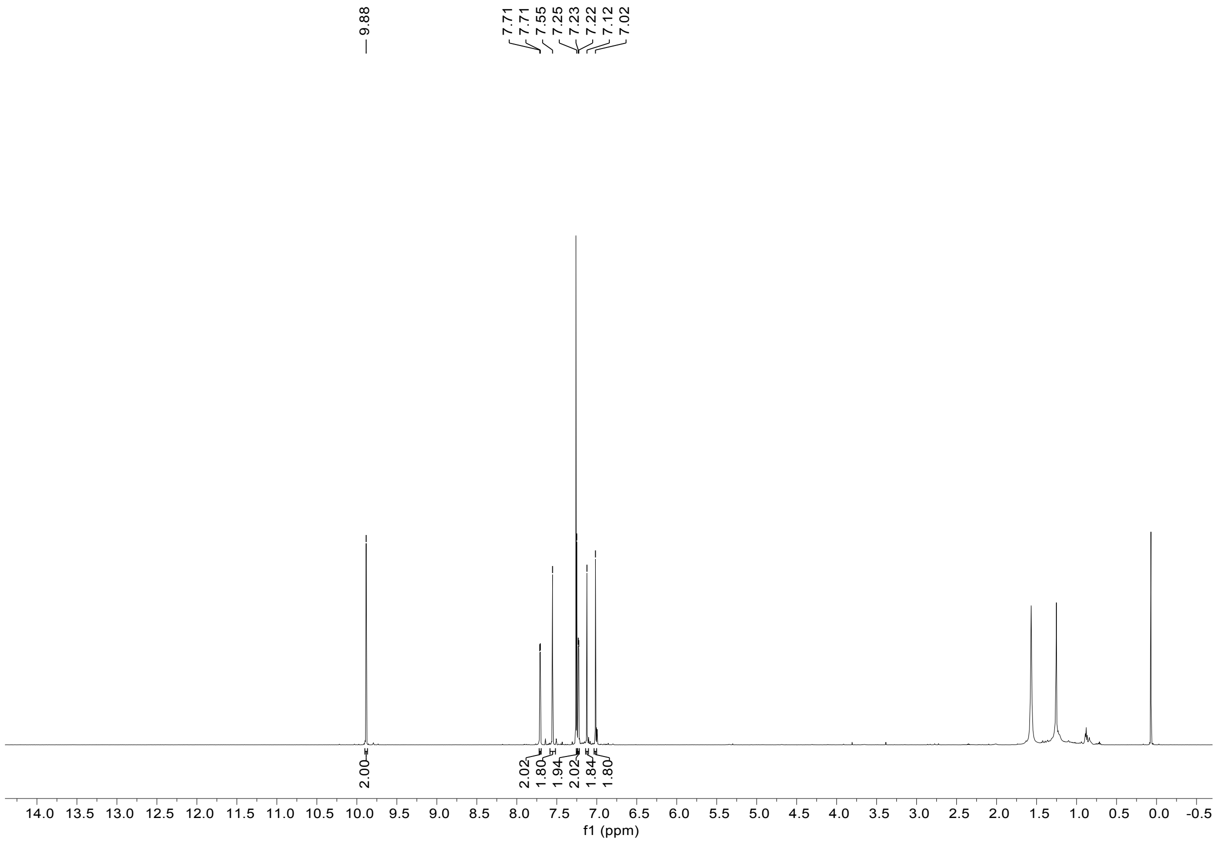

Figure S27. ^{1}H NMR spectrum of compound **8**.

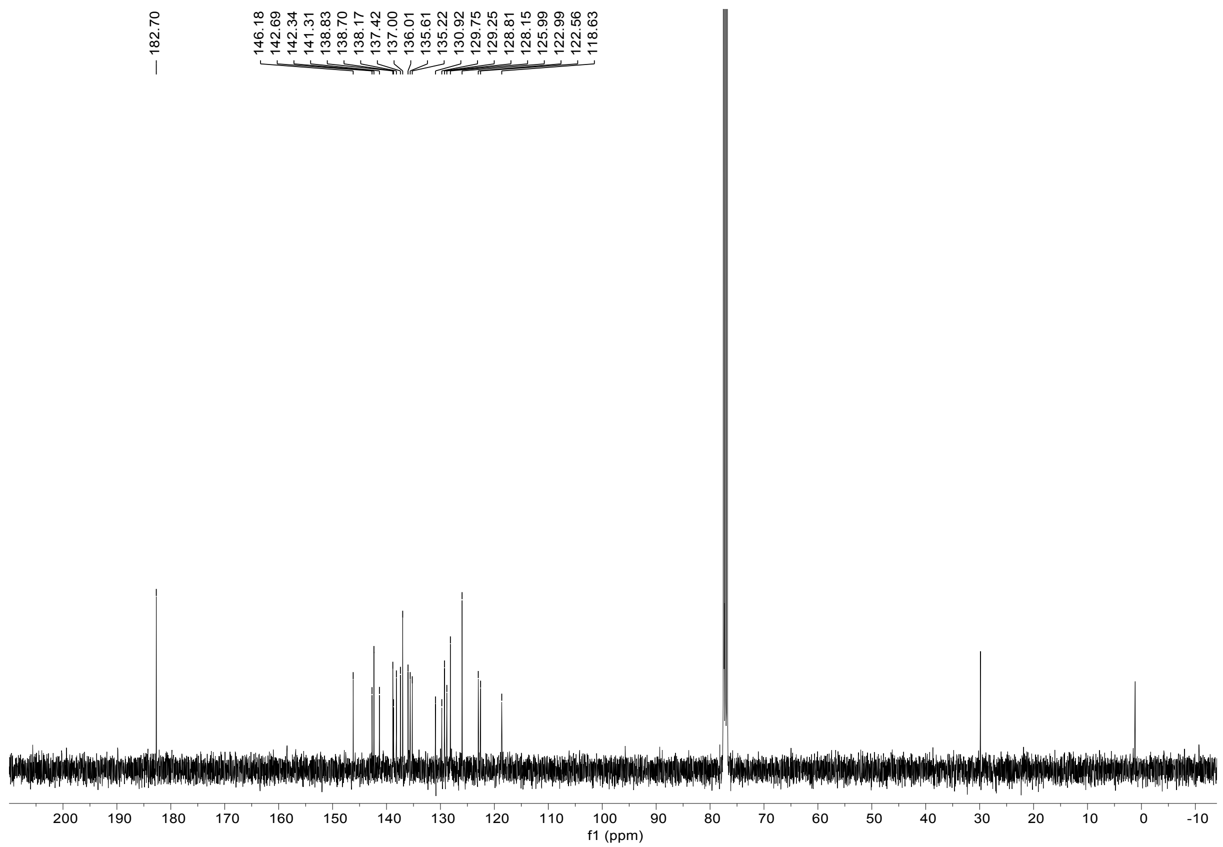

Figure S28. ^{13}C NMR spectrum of compound **8**.

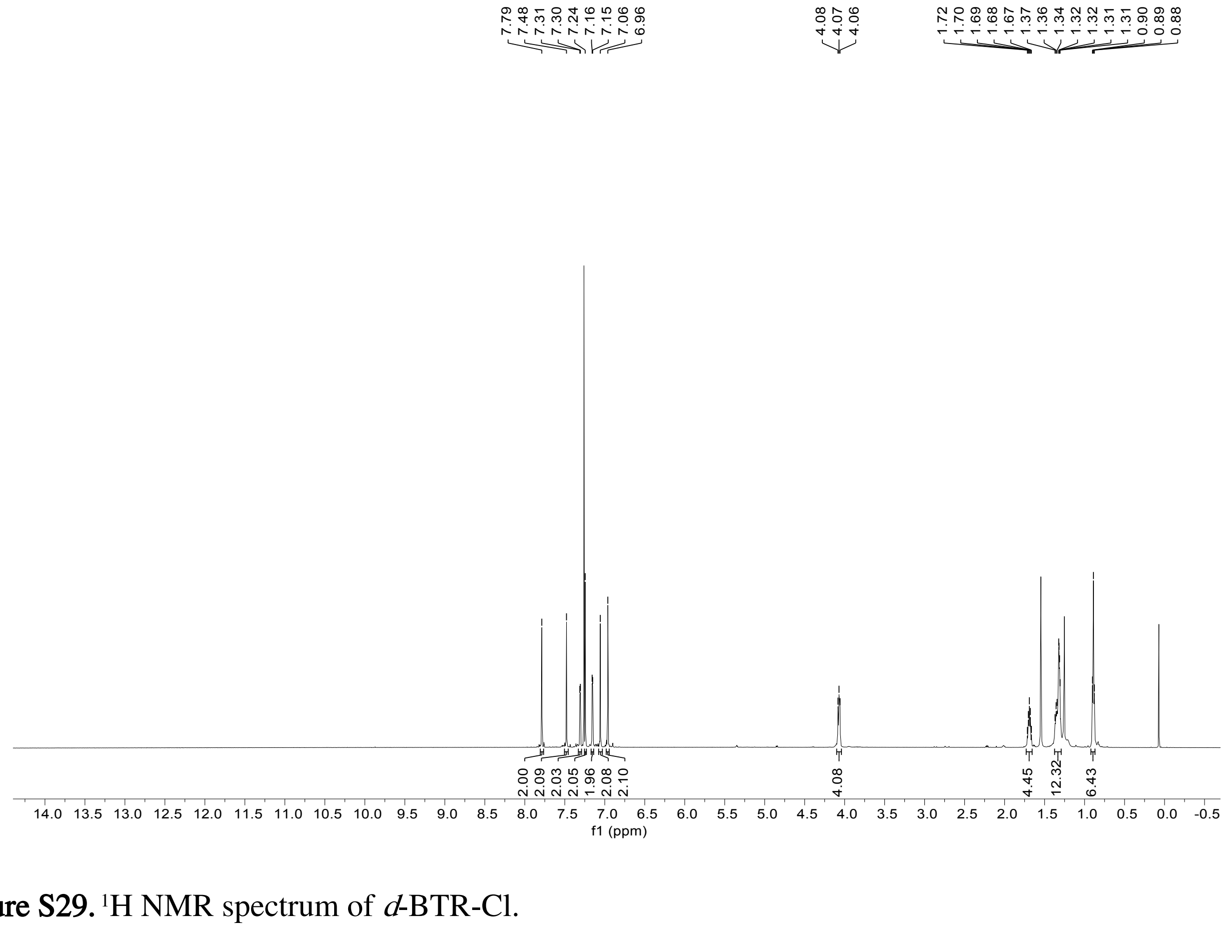

Figure S29. ^{1}H NMR spectrum of *d*-BTR-Cl.

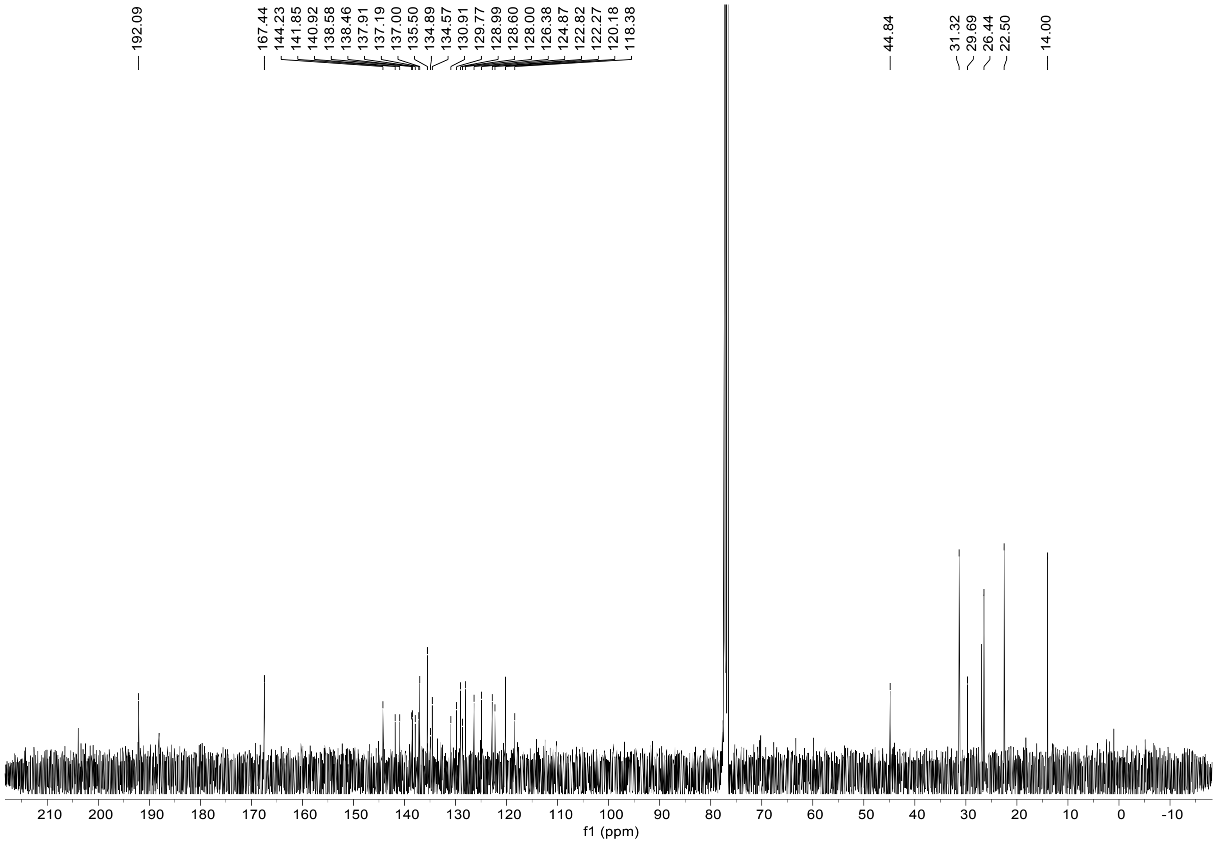

Figure S30. ^{13}C NMR spectrum of *d*-BTR-Cl.

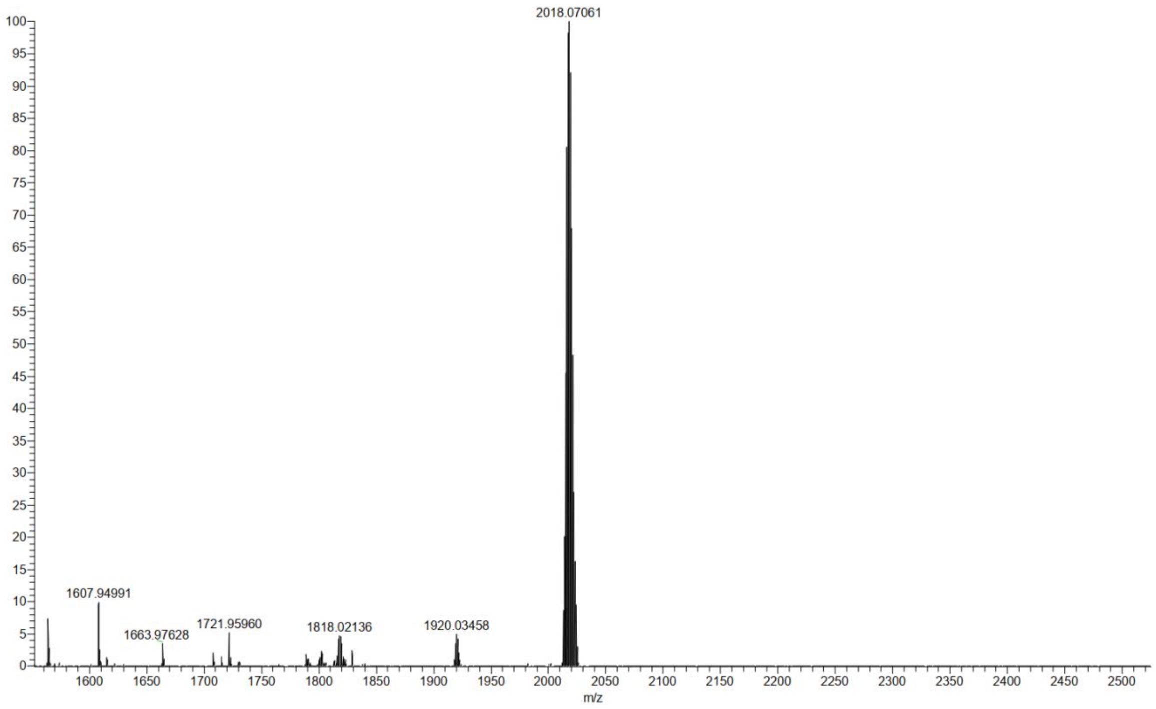

Figure S31. Mass spectrometry of d-BTR-Cl.

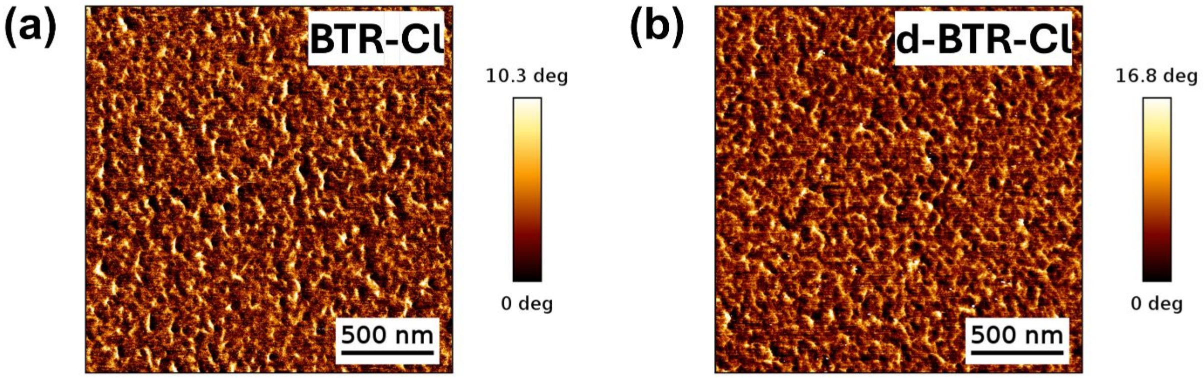

Figure S32. Phase image of BTR-Cl (a) and d-BTR-Cl (b).

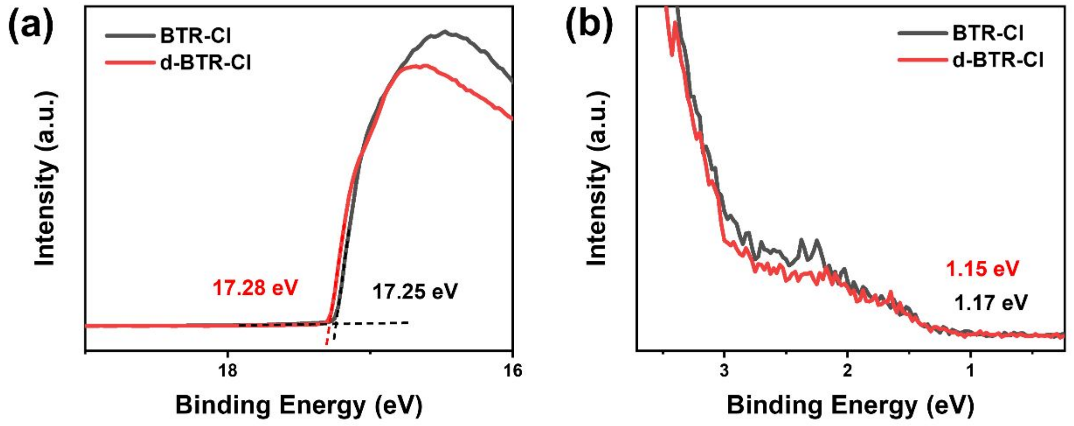

Figure S33. UPS spectra of BTR-Cl and d-BTR-Cl films. (a) Secondary electron cutoff region for determining the work function. (b) Valence band region for determining the ionization energy.

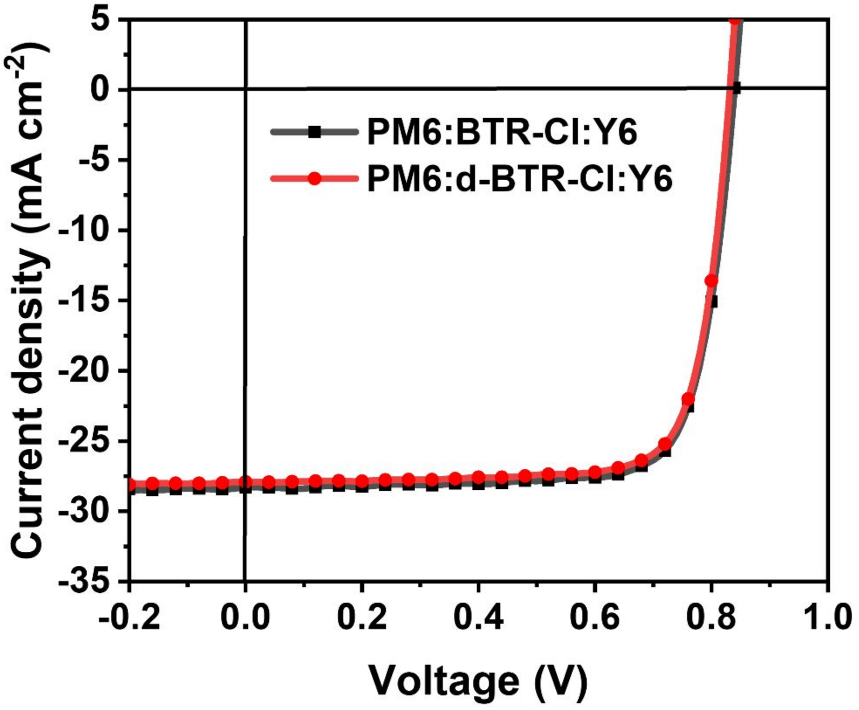

Figure S34. J-V curves of PM6:BTR-Cl:Y6 and PM6:d-BTR-Cl:Y6.

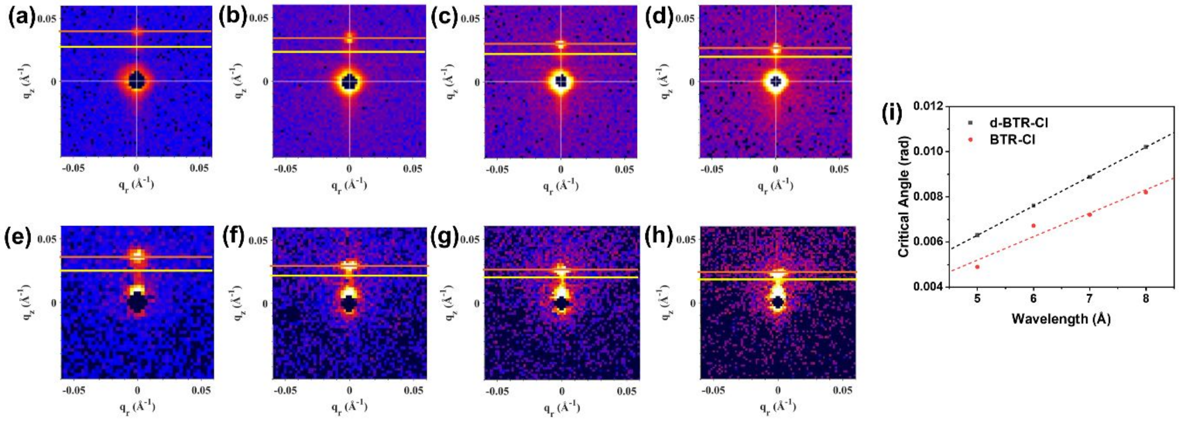

Figure S35. TOF-GISANS measurements of pure films. (a-d) d-BTR-Cl measured at different wavelengths of a-d 5-8 Å. (e-h) BTR-Cl measured at different wavelengths of a-d 5-8 Å. (i) The

extracted critical angles of d-BTR-Cl films as a function of neutron wavelengths (dots) with the best linear fits (dashed lines)

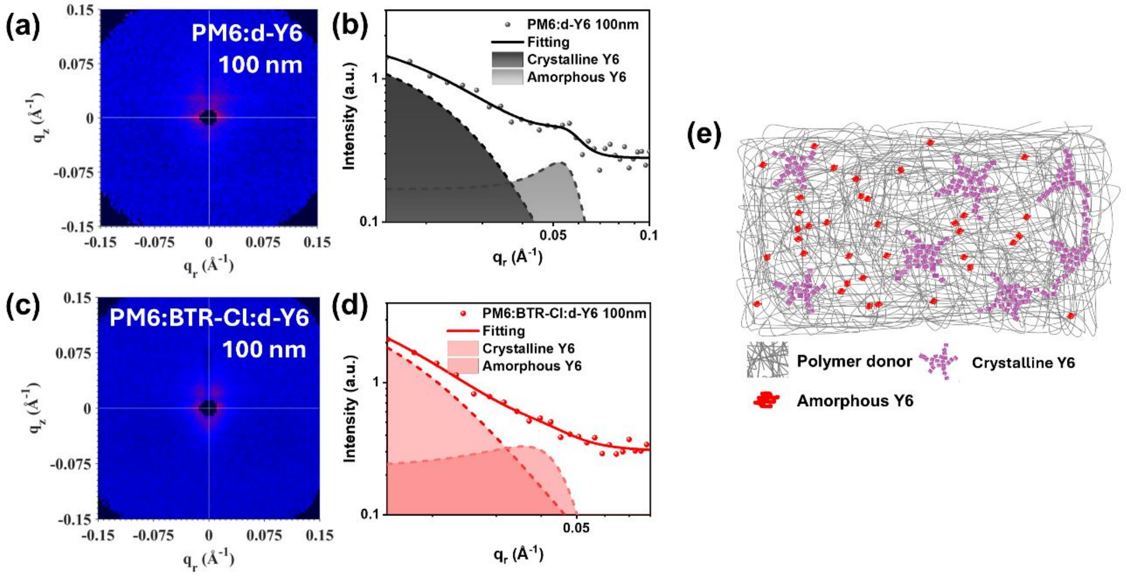

Figure S36. TOF-GISANS measurements of 100 nm blend films. (a,c) TOF-GISANS patterns of PM6:d-Y6 and PM6:BTR-Cl:d-Y6 with their horizontal linecuts (dots) with best fits (solid lines) shown in (b) and (d), respectively. The schematics (e) show the main features that give rise to scattering contrasts in GISANS measurements

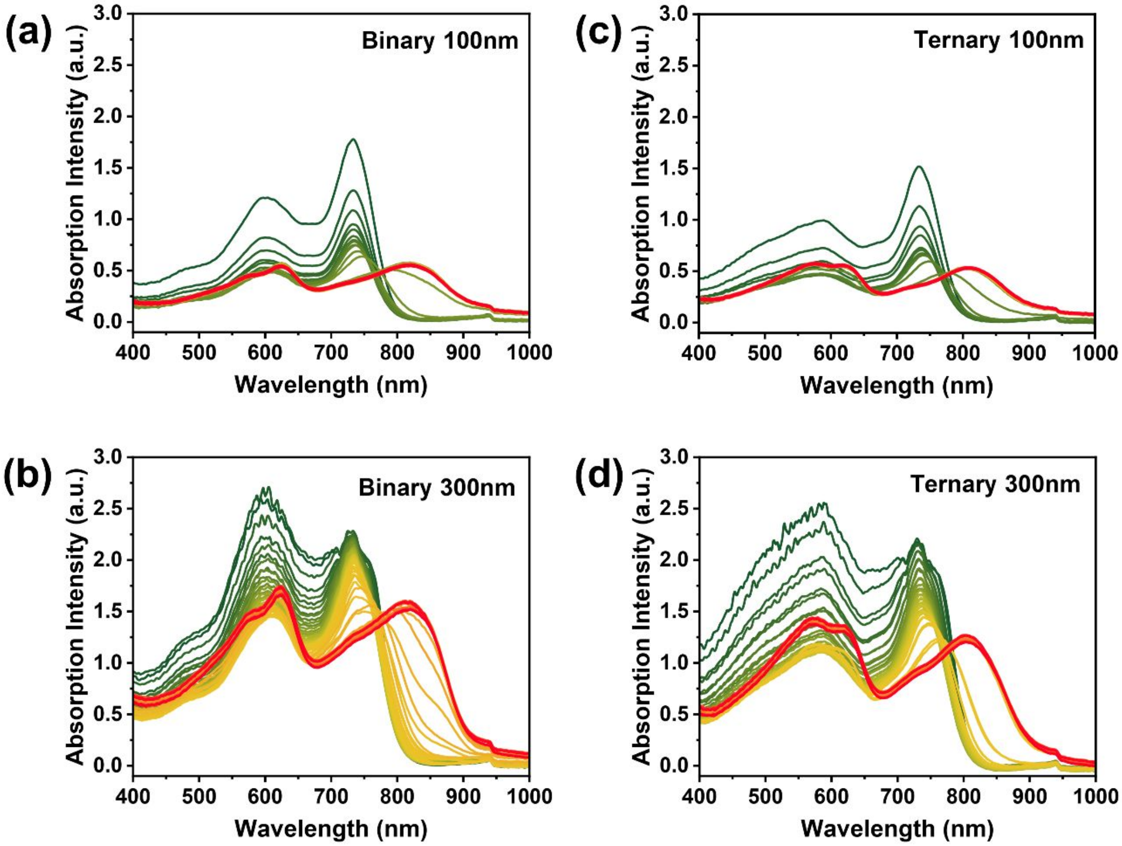

Figure S37. In-situ UV-vis absorption spectra for binary and ternary blend films with different thickness. (a) Binary 100 nm, (b) Binary 300 nm, (c) Ternary 100 nm, and (d) Ternary 300 nm.

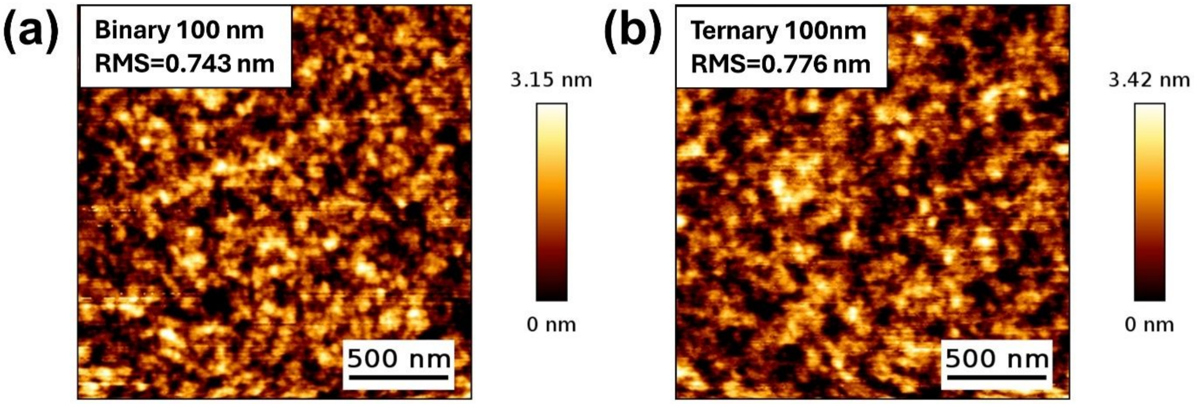

Figure S38. AFM topography images for 100nm binary (a) and ternary (b) blend films.

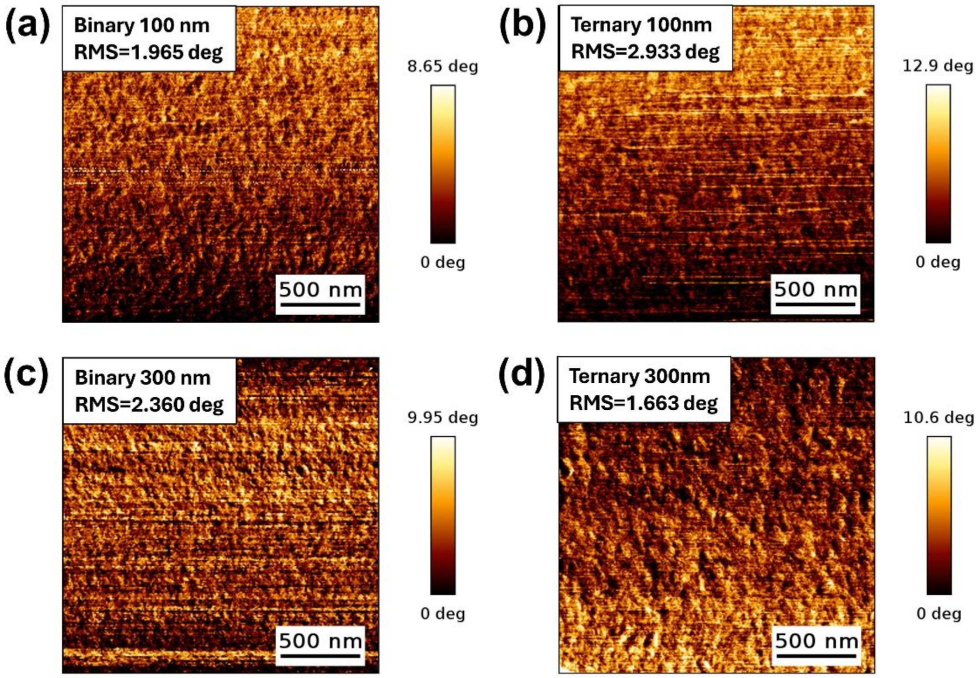

Figure S39. AFM phase images for binary and ternary blends with different thickness. (a) Binary 100 nm, (b) Ternary 100 nm, (c) Binary 300 nm, and (d) Ternary 300 nm.

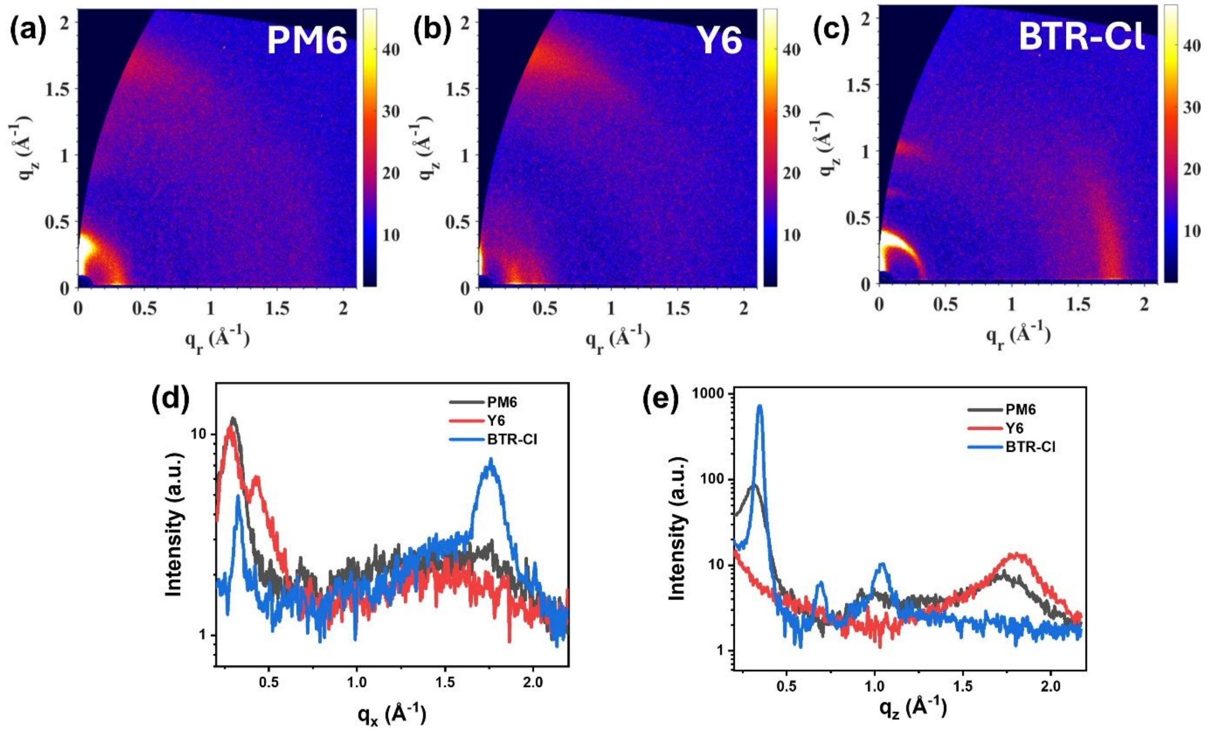

Figure S40. (a-c) GIWAXS 2D patterns of neat films and (d-e) corresponding linecuts in q_r and q_z direction.

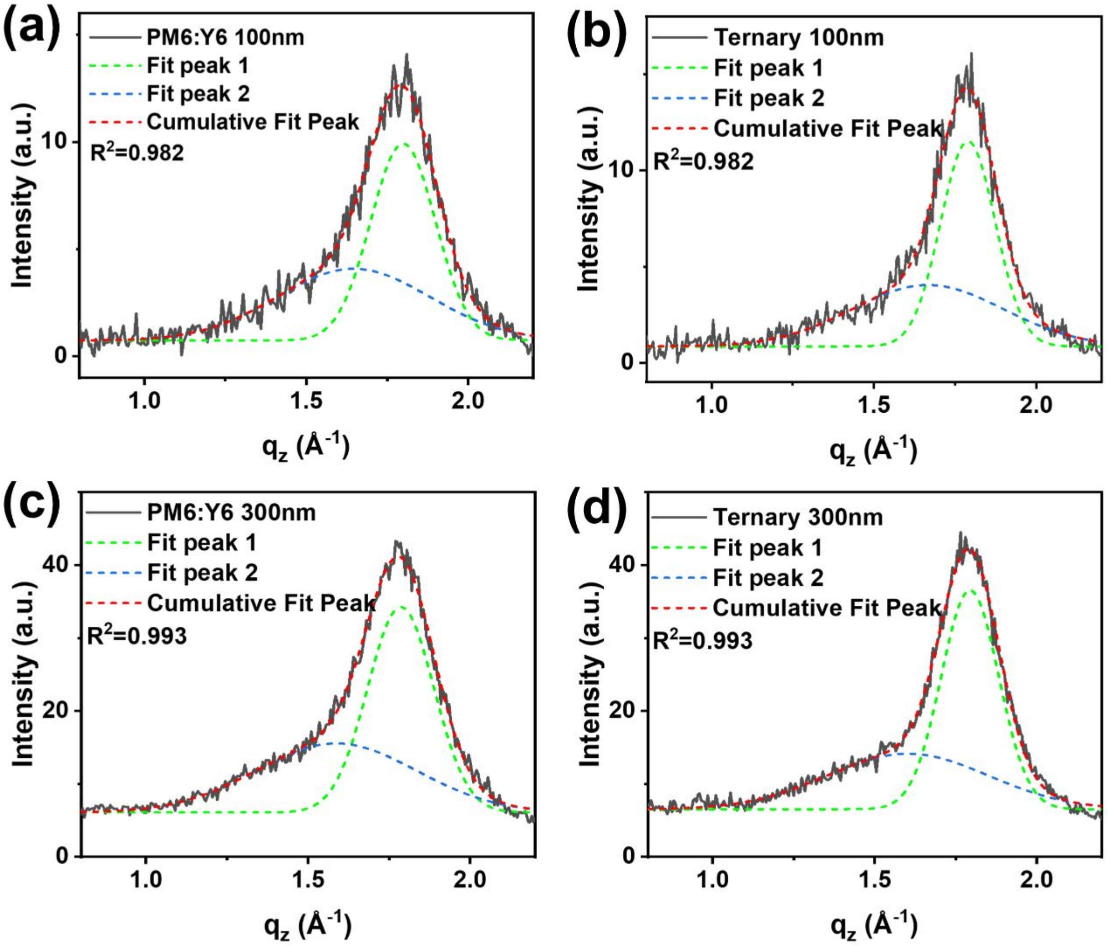

Figure S41. The de-convolution of different blend film π−π peaks. (a) Binary 100 nm, (b) Ternary 100 nm, (c) Binary 300 nm, and (d) Ternary 300 nm.

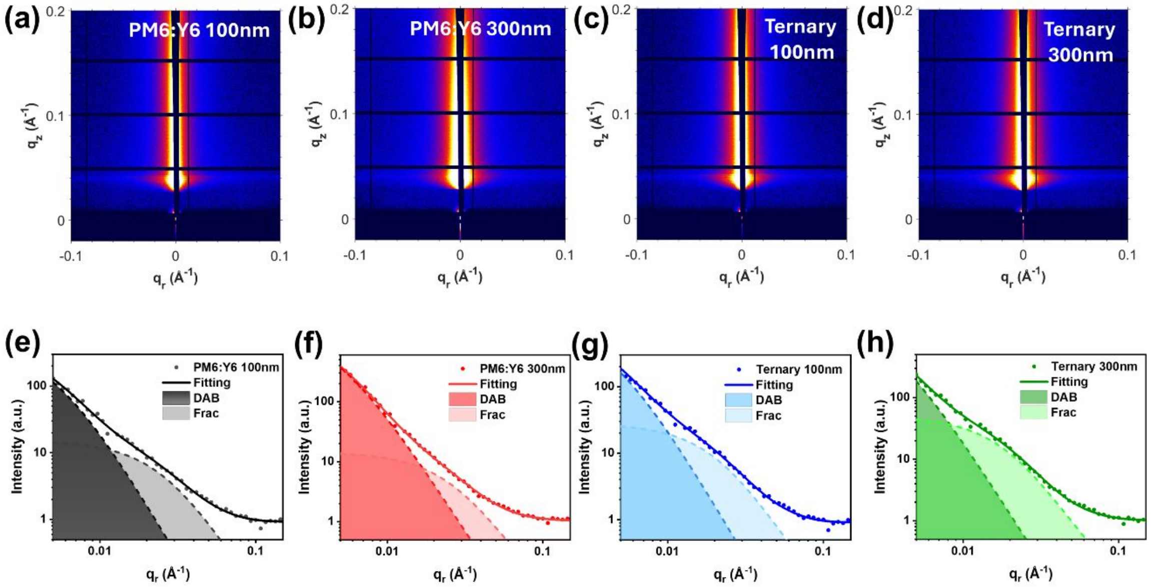

Figure S42. The GISAXS 2D pattern (a-d) and the horizontal linecuts (e-h) with best fits of thin and thick blend film with different D/A ratios.

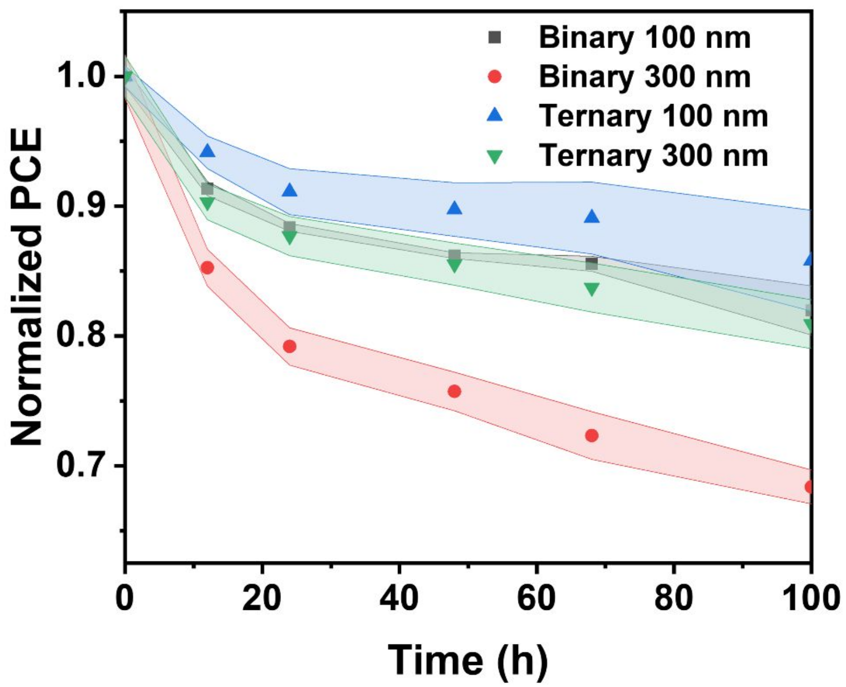

Figure S43. Normalized PCE retention of binary and ternary OSCs with 100 nm and 300 nm active layer thicknesses over 100 h under nitrogen conditions.

Table S1. Performance of the optimized 400 nm OSCs under illumination of AM 1.5 G, 100 mW cm^{-2}

Additive	V_{OC} (V)	PCE(%)	Fill Factor (%)	J_{SC} (mA/cm^2)
Binary 400nm	0.806	10.0 (9.4±0.5)	52.2	23.7
Ternary 400nm	0.819	15.9 (14.6±0.7)	65.1	29.9

Table S2. Charge Mobilities of Blend Films Measured by SCLC Method

Active layer	μ_h (× 10^{-4} cm^2 V^{-1} s^{-1})	μ_e (× 10^{-4} cm^2 V^{-1} s^{-1})	μ_h/μ_e
Binary 100nm	7.3	5.4	1.35
Ternary 100nm	12.9	13.0	0.99
Binary 300nm	4.9	2.1	2.33
Ternary 300nm	10.8	9.9	1.09

Table S3. GISANS fitting results.

	Crystalline Y6 (nm)	Amorphous Y6 (nm)	Crystalline BTR-Cl (nm)	Amorphous BTR-Cl (nm)
PM6:d-Y6 100nm	16.7	8.7	N/A	N/A
PM6:BTR-Cl:d-Y6 100nm	26.2	10.3	N/A	N/A
PM6:d-Y6 300nm	21.9	N/A	N/A	N/A
PM6:BTR-Cl:d-Y6 300nm	26.6	9.1	N/A	N/A
PM6:d-BTR-Cl:Y6 300nm	N/A	N/A	16.8	9.7

Table S4. Detailed parameters for c-AFM images.

	Average value (pA)	Ra (pA)	RMS (pA)
Binary 300nm	-215.5	32.96	43.90
Ternary 300nm	-314.9	43.22	59.13

Table S5. Calculated GIWAXS parameters of blend films.

q_z	π-π stacking		FWHM	CCL
	Peak ($Å^{-1}$)	d-spacing (Å)		
Binary 100nm	1.80	3.49	0.236	23.95
Binary 300nm	1.79	3.51	0.241	23.45
Ternary 100nm	1.79	3.51	0.198	28.55
Ternary 300nm	1.79	3.51	0.210	26.91

Table S6. Calculated GIWAXS characteristics of neat films in q_z direction.

q_z	Lamellar		π-π stacking	
	Peak ($Å^{-1}$)	d-spacing (Å)	Peak ($Å^{-1}$)	d-spacing (Å)
PM6	0.310	20.3	1.74	3.61
Y6	/	/	1.80	3.49
BTR-Cl	0.346	18.2	/	/

Table S7. Calculated GIWAXS characteristics of neat films in q_r direction.

q_r	Lamellar		π-π stacking	
	Peak (Å^{-1})	d-spacing (Å)	Peak (Å^{-1})	d-spacing (Å)
PM6	0.300	20.9	/	/
Y6	0.279	20.5	/	/
BTR-Cl	0.325	19.3	1.76	3.57

Table S8. GISAXS fitting results.

	DAB (nm)	$2R_g$ (nm)
PM6:Y6 100nm	19.6	16.9
PM6:Y6 300nm	27.8	21.9
PM6:BTR-Cl:Y6 100nm	20.3	18.8
PM6:BTR-Cl:Y6 300nm	23.1	18.9
